# Early Steps in Autophagy Depend on Direct Phosphorylation of Atg9 by the Atg1 Kinase

**DOI:** 10.1016/j.molcel.2013.12.011

**Published:** 2014-02-06

**Authors:** Daniel Papinski, Martina Schuschnig, Wolfgang Reiter, Larissa Wilhelm, Christopher A. Barnes, Alessio Maiolica, Isabella Hansmann, Thaddaeus Pfaffenwimmer, Monika Kijanska, Ingrid Stoffel, Sung Sik Lee, Andrea Brezovich, Jane Hua Lou, Benjamin E. Turk, Ruedi Aebersold, Gustav Ammerer, Matthias Peter, Claudine Kraft

**Affiliations:** 1 Max F. Perutz Laboratories, University of Vienna, 1030 Vienna, Austria; 2 Department of Biology, Institute of Molecular Systems Biology, ETH Zürich, Wolfgang Pauli Strasse 16, 8093 Zürich, Switzerland; 3 Institute of Biochemistry, Department of Biology, ETH Zürich, Schafmattstrasse 18, 8093 Zürich, Switzerland; 4 Department of Pharmacology, Yale University School of Medicine, New Haven, CT 06520, USA

## Abstract

Bulk degradation of cytoplasmic material is mediated by a highly conserved intracellular trafficking pathway termed autophagy. This pathway is characterized by the formation of double-membrane vesicles termed autophagosomes engulfing the substrate and transporting it to the vacuole/lysosome for breakdown and recycling. The Atg1/ULK1 kinase is essential for this process; however, little is known about its targets and the means by which it controls autophagy. Here we have screened for Atg1 kinase substrates using consensus peptide arrays and identified three components of the autophagy machinery. The multimembrane-spanning protein Atg9 is a direct target of this kinase essential for autophagy. Phosphorylated Atg9 is then required for the efficient recruitment of Atg8 and Atg18 to the site of autophagosome formation and subsequent expansion of the isolation membrane, a prerequisite for a functioning autophagy pathway. These findings show that the Atg1 kinase acts early in autophagy by regulating the outgrowth of autophagosomal membranes.

## Highlights

• The Atg1 kinase phosphorylation consensus was identified on peptide arrays
• Atg9 is a direct target of the Atg1/ULK1 kinase in vitro and in vivo
• Atg9 phosphorylation recruits Atg18 and Atg8 to the PAS
• Atg9 phosphorylation is required for isolation membrane expansion/autophagy function

## Graphical Abstract

**Figure:**
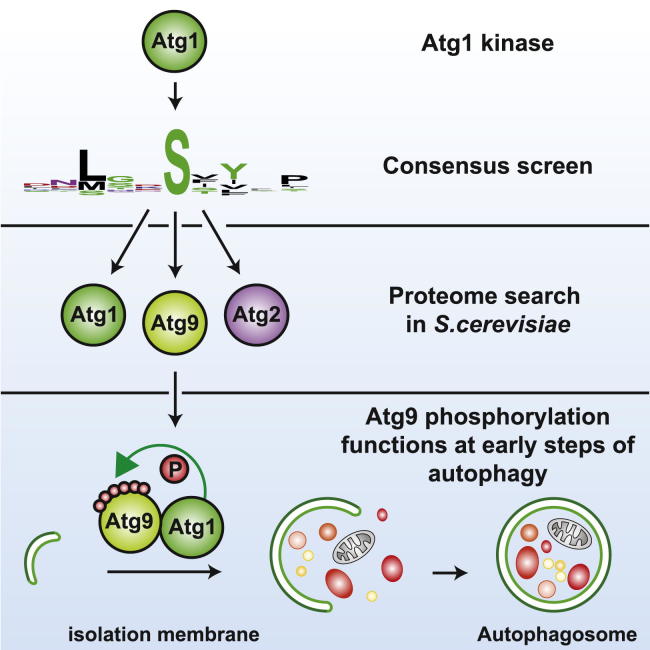

## Teaser

Autophagy function is pivotal to cell health. Papinski et al. identify the phosphorylation consensus of the central kinase in this pathway, Atg1. The autophagy-related protein Atg9 is a direct target of Atg1. Atg9 phosphorylation by Atg1 is required for autophagosome formation. This finding sheds light on how Atg1 controls autophagy.

## Introduction

Macroautophagy, hereafter referred to as autophagy, is an important cellular mechanism for the bulk degradation of cytoplasmic material. Autophagy is a sequential process beginning with the sequestration of cytoplasmic cargo in an expanding membrane sac known as the phagophore, which forms at the preautophagosomal structure (PAS). The phagophore expands to form a double-membrane autophagosome that engulfs the cargo and subsequently fuses with the lysosome or vacuole where the cargo is degraded. This conserved process serves to ensure cellular homeostasis, and is integral for the cellular response to stress conditions such as nutrient starvation as well as in embryonic development and the defense against several human pathogens. Indeed, defects in autophagy pathways have been associated with numerous human pathologies including infectious diseases, neurodegenerative disorders, and cancer. Despite these fundamental functions, the molecular mechanisms underlying autophagy regulation remain poorly understood.

Both nonselective “bulk” autophagy and selective autophagy of specific proteins or organelles have been described (Kraft et al., 2009). A selective autophagy-related pathway in yeast is the cytoplasm-to-vacuole targeting (Cvt) pathway, which fulfills a biosynthetic function by delivering three resident enzymes, aminopeptidase 1 (Ape1), α-mannosidase (Ams1), and aspartyl aminopeptidase (Ape4), to the vacuole (Harding et al., 1995; Hutchins and Klionsky, 2001; Yuga et al., 2011). Genetic analysis in yeast has identified 36 mostly conserved components (termed Atg1–Atg36) that are required for different steps of autophagy (Motley et al., 2012; Suzuki and Ohsumi, 2007). Many of these components are common to both autophagy and the Cvt pathway, although autophagy- and Cvt-specific genes exist (Inoue and Klionsky, 2010). Atg1 is a serine-threonine kinase required for both selective and bulk autophagy pathways, and it is highly conserved from yeast to mammals. A single homolog of Atg1 has been identified in *Caenorhabditis elegans* and *Drosophila melanogaster*, and the homologs Unc-51-like kinase 1 (ULK1) and ULK2 (Meléndez et al., 2003; Scott et al., 2007; Tomoda et al., 1999) have been identified in mammals. The kinase activity of Atg1 and its homologs is needed for autophagy in mammalian cells (Hara et al., 2008), and for both autophagy and the Cvt pathway in yeast (Matsuura et al., 1997).

Among proteins essential for autophagy is the conserved multimembrane-spanning protein Atg9 (Lang et al., 2000; Noda et al., 2000). A role for Atg9 in the regulation of autophagy by providing membranes for autophagosome formation in yeast and possibly also in mammals has been suggested (Mari et al., 2010; Orsi et al., 2012; Yamamoto et al., 2012). Atg9 localizes to several vesicles throughout the cytoplasm, and shuttling of Atg9 to and from the PAS has been proposed to be important for autophagy function (Reggiori et al., 2004). Atg9 vesicles are 30–60 nm in diameter and contain approximately 30 molecules of Atg9 (Yamamoto et al., 2012). These vesicles fuse to form the preautophagosomal structure, and subsequently Atg9 becomes integrated into the outer autophagosomal membrane. After fusion of autophagosomes with the vacuole, Atg9 is recycled. Several factors including Atg1, Atg2, and Atg18 have been implicated in the regulation of Atg9 (Reggiori et al., 2004).

Despite the important role of Atg1 in autophagy, little is known about its physiological substrates for autophagy function. Although some Atg1 kinase targets have been described in *D. melanogaster* and mammalian cells (Chen et al., 2008; Di Bartolomeo et al., 2010; Russell et al., 2013; Tang et al., 2011), the mechanism by which Atg1 kinase regulates autophagy remains elusive. Understanding the detailed events of how Atg1 phosphorylation regulates autophagy and identification of its crucial substrates for autophagy function is key to deciphering the mechanisms of autophagy induction during nutrient starvation and other stress conditions.

In this study, we set out to identify Atg1 kinase substrates in yeast. We used a combination of peptide array-based consensus screening and quantitative mass spectrometry to deduce Atg1 kinase substrates. Among 24 confirmed phosphorylation targets, this approach identified Atg9 as a direct kinase substrate of Atg1. Indeed, mutation of the Atg1-dependent phosphorylation sites on Atg9 resulted in severe defects in both selective and bulk autophagy. Although nonphosphorylatable Atg9 mutants shuttle normally in the cell, they fail to interact with and recruit the essential autophagy proteins Atg18 and Atg8 to the PAS, resulting in the inability to form autophagosomal isolation membranes. Together, these findings identify Atg9 as a direct Atg1 kinase target essential for autophagy function in yeast, and unravel a mechanistic role for Atg1 at the PAS early during autophagosome formation.

## Results

### Determination of the Atg1 Kinase Consensus Phosphorylation Site Motif

**Figure 1:**
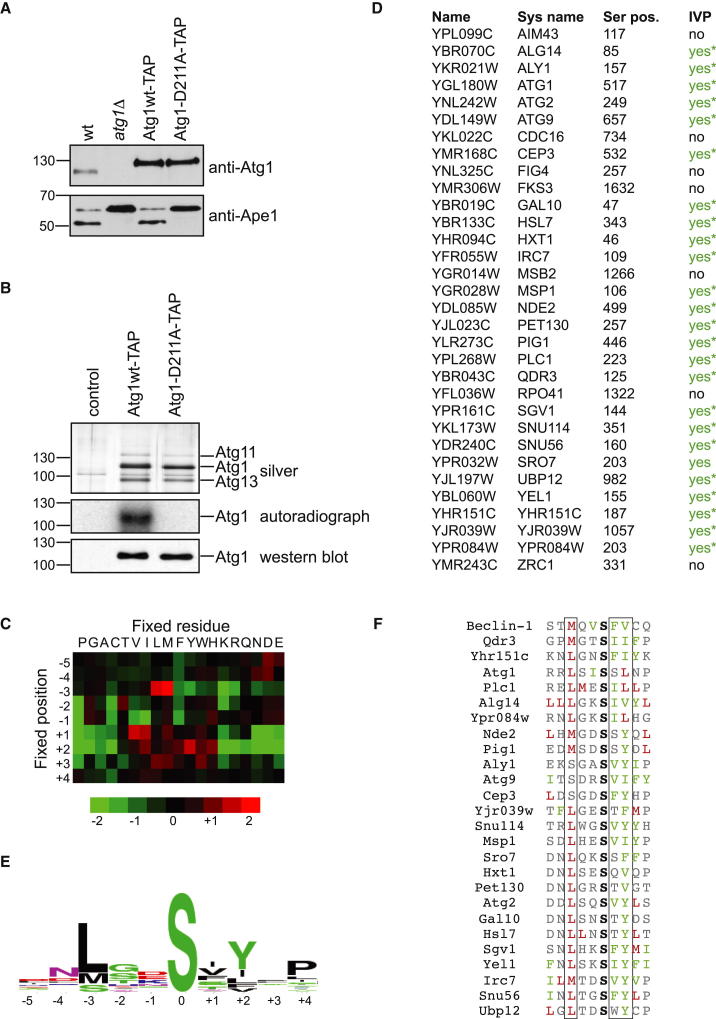
Identification of the Atg1 Kinase Consensus Phosphorylation Site (A) Wild-type (wt), *atg1Δ*, and genomically integrated TAP-tagged wild-type Atg1 (wt) or Atg1 kinase-dead D211A mutant cells were grown to mid-log phase. Cell extracts were prepared by trichloroacetic acid precipitation and analyzed by anti-Atg1 and anti-Ape1 western blotting. Note that TAP-tagged constructs seem more abundant than untagged Atg1 due to additional antibody binding to protein A in western blotting. (B) Wild-type (control), genomically integrated TAP-tagged wild-type Atg1 (Atg1wt), or Atg1 kinase-dead D211A mutant cells were grown to mid-log phase and rapamycin treated for 1 hr. Atg1 was immunoprecipitated and eluted by TEV cleavage. The eluate was analyzed by anti-Atg1 western blotting and silver staining, and autophosphorylation activity was assessed in vitro. The identity of bands corresponding to Atg1, Atg11, and Atg13 on the silver gels was confirmed by mass spectrometry. (C) Peptides (16-mer) containing a central fixed serine phosphoacceptor flanked by degenerate positions and one fixed position as indicated were phosphorylated in vitro using soluble Atg1 complexes, and reactions were spotted on streptavidin-coated membranes followed by autoradiographic quantification. A heat map representing the quantified peptide array is shown. (D) The quantified consensus motif obtained from the peptide array was searched against the *S. cerevisiae* protein database. Synthetic peptides spanning the consensus region of the top 30 hits and the consensus region of Atg1 and Atg9 were generated and subjected to in vitro phosphorylation with soluble wild-type Atg1 and the D211A mutant, followed by mass spectrometric identification of their phosphorylation state (IVP). Peptides found to be in vitro phosphorylated are marked with “yes.” Asterisks mark accumulation of precursor ion intensity over time, shown in Figure S5. (E) A consensus logo was generated by aligning the 25 verified consensus hits using WebLogo (http://weblogo.berkeley.edu) (Crooks et al., 2004). (F) Alignment of the 25 in vitro verified Atg1 kinase targets with the known ULK1 substrate Beclin-1. M and L are marked in red (position −3 according to the peptide array), and F, V, I, and Y are in green (positions +1 and +2). Note that S can also be found at position −3 and L at position +2. See also Figures S1 and S5.

Atg1 kinase activity is essential for autophagy function; however, phosphorylation targets have remained sparse. To identify Atg1 kinase targets relevant for autophagy, we mapped the Atg1 kinase consensus phosphorylation site motif using a peptide array. Previous large-scale studies deciphering kinase consensus site motifs used overexpression of the kinase of interest (Mok et al., 2010). However, as Atg1 overexpression is toxic in yeast and Atg1 is found in a large protein complex together with several proteins (including its essential activator Atg13), we purified endogenously expressed Atg1 to preserve the native complex and interactions, a prerequisite for the identification of physiological substrates. Protein A-tagged Atg1 was shown to be fully functional as determined by cleavage of the propeptide from the preenzyme Ape1 (prApe1) to generate its active form in the vacuole (Figure 1A) (Klionsky et al., 1992; Kraft et al., 2012). After induction of autophagy by rapamycin treatment, the Atg1 complex was affinity purified from yeast cultures and released from the beads by tobacco etch virus (TEV) protease cleavage (Figure 1B). The identity of Atg1, Atg11, and Atg13 was verified by mass spectrometric analysis of the respective silver-stained gel bands. The activity of the resulting soluble complex was then analyzed in an in vitro autophosphorylation reaction (Figure 1B; Figure S1A available online). Whereas the wild-type protein was active, the kinase-dead Atg1 D211A mutant did not show in vitro autophosphorylation activity, indicating the absence of copurified kinase activity. To identify the preferred phosphorylation motif, this soluble kinase was assayed in peptide arrays as described previously (Mok et al., 2010). The spot intensities from the peptide array were quantified, background corrected, and normalized. These values were used to build heat maps indicating the preference for each amino acid type at the respective position relative to the serine phosphorylation acceptor (Figure 1C). Atg1 had a unique phosphorylation motif characterized by preferences for hydrophobic residues at multiple positions. For example, Atg1 was most selective for aliphatic residues at position −3 and for both aliphatic and aromatic residues at positions +1 and +2, and there was strong negative selection against small and charged residues at these positions. In addition, Atg1 showed a clear preference for serine over threonine as the phosphoacceptor residue.

### Validation of Atg1 Kinase Substrates

A position-specific scoring matrix of selectivity values was used to search the *Saccharomyces cerevisiae* protein sequence database for sites closely matching the Atg1 kinase consensus sequence (Obenauer et al., 2003). Among these potential Atg1 kinase substrates, the autophagy protein Atg2 was identified (Figure 1D). We furthermore noted that Atg1 and Atg9 also contain Atg1 consensus sites, and included these in the subsequent analysis. To verify that the identified candidate substrates are bona fide Atg1 kinase targets, we synthesized 32 peptides spanning the consensus regions of the candidate proteins and incubated them with soluble Atg1 kinase in the presence of ATP. The resulting phosphorylated peptides were then analyzed by shotgun mass spectrometry. As in vitro kinase reactions can generate nonspecific phosphorylation artifacts, we controlled for both Atg1 phosphorylation specificity and for incorrect phosphopeptide identifications by sampling the reaction at 5, 30, and 60 min and monitoring the accumulation of phosphorylation over time by label-free quantification using the precursor ion intensities of the respective peptides. For most of the identified phosphorylation sites, accumulation of precursor ion intensities could be detected over time (Figure 1D, marked with asterisks; Figure S5), further indicating that the in vitro phosphorylation events were specifically associated with the Atg1 complex. In addition, experiments performed with the kinase-dead mutant of Atg1 did not generate any phosphorylated peptides. This confirms that the kinase activity detected is dependent only on Atg1 and not any other copurifying enzyme. Out of 32 candidates tested, 25 were verified in vitro as direct Atg1 kinase substrates, validating our approach. A refined consensus sequence was generated by the comparison of the 25 substrates, illustrated by a WebLogo cartoon (Figure 1E) (Crooks et al., 2004). Alignment of the 25 verified Atg1 kinase targets with the known mammalian ULK1 target Beclin-1 showed that the consensus preference is indeed conserved from yeast to mammals (Figure 1F) (Russell et al., 2013). We focused our follow-up analysis on the known autophagy players Atg1, Atg2, and Atg9, as in budding yeast no Atg1 kinase substrate has been identified to date.

### Atg1, Atg2, and Atg9 Are In Vivo Atg1 Kinase Targets

To determine whether Atg1, Atg2, and Atg9 are phosphorylated in an Atg1-dependent manner in vivo, we analyzed the phosphorylation sites of these proteins by mass spectrometric stable isotope labeling by amino acids in cell culture (SILAC) quantification. We purified Atg1, the kinase-dead Atg1 D211A mutant, Atg2, and Atg9 from rapamycin-treated wild-type and *atg1Δ* cells under denaturing conditions using a C-terminally integrated His-biotin (HTB) tag (Kijanska et al., 2010; Reiter et al., 2012). These constructs are fully functional, as judged by prApe1 processing (Figure S1B) (Kijanska et al., 2010).

Eleven autophosphorylation sites on Atg1 and four Atg1-regulated phosphorylation events on Atg2 were detected (Figures S1C and S1E). The predicted phosphorylation sites S517 on Atg1 and S249 on Atg2 were found phosphorylated in vivo, strongly supporting that these are bona fide Atg1 phosphorylation sites. In addition, S356 and S390 on Atg1 matched the consensus sequence, further indicating that these are also autophosphorylation sites of Atg1 (Figure S1D).

In addition to S249, S1086 on Atg2 also conforms to the Atg1 consensus (Figure S1F). Thus, we mutated S249 and S1086 on Atg2 to nonphosphorylatable alanine or phospho-mimicking aspartate and tested their functionality by measuring prApe1 processing. Neither mutation had an effect on prApe1 maturation (Figure S1G), suggesting that these phosphorylation sites act in a more specialized autophagy pathway and/or serve redundant functions.

**Figure 2:**
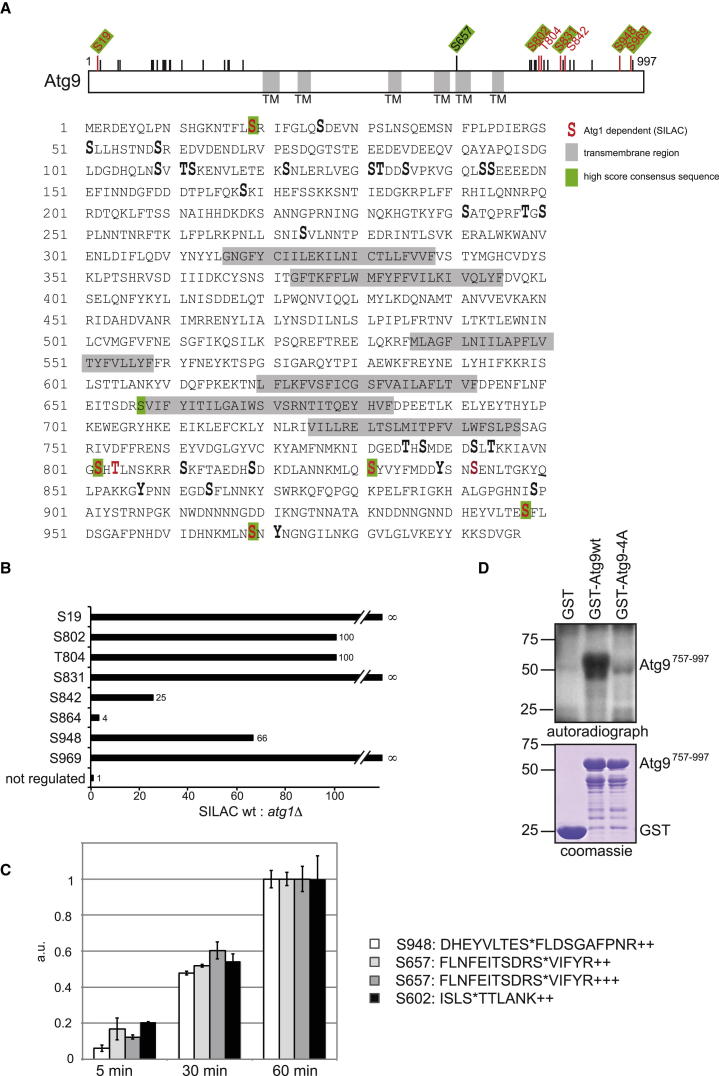
Atg9 Is an Atg1 Kinase Target In Vivo and In Vitro (A) Wild-type and *atg1Δ* yeast cells containing endogenously tagged Atg9-HTBeaq were grown to mid-log phase and rapamycin treated for 45 min. Atg9 was affinity purified and subjected to quantitative mass spectrometric phosphorylation mapping by SILAC. Phosphorylation sites are shown enlarged and bold, Atg1-dependent sites are indicated in red, and sites corresponding to the Atg1 consensus are shaded in green. Gray indicates a transmembrane region (TM). (B) Ratios of wild-type (13C/heavy) versus *atg1Δ* (12C/light) are shown. ∞ indicates that only the heavy variant of the peptide was detected. (C) Synthetic peptides spanning Atg9 (Table S4) were generated and subjected to in vitro phosphorylation with soluble wild-type Atg1 and the D211A mutant, followed by mass spectrometric identification of their phosphorylation state. Peptides that were phosphorylated by wild-type Atg1 in vitro are quantified over the time course of the reaction (0, 5, 30, and 60 min) by label-free quantification using the precursor ion intensities of specific peptides. Note that two different peptides spanning S657 were identified. a.u., arbitrary units. The intensities from the MS1 filtering were normalized to the values from the 60 min reactions. Error bars represent normalized standard deviation of the replicate injections. (D) The C-terminal fragment encompassing amino acids 757–997 of Atg9 or the corresponding 4A mutant (S802A, S831A, S948A, S969A) was expressed in *E. coli* with a glutathione S-transferase (GST) tag, immobilized on beads, and in vitro phosphorylated with soluble Atg1. Proteins were analyzed by Coomassie staining, and radioactive incorporation was assessed by autoradiography. Note that all highly regulated Atg1 consensus sites are mutated in this 4A mutant, as S19 and S657 are not part of the C-terminal fragment. See also Figures S2, S5, and Table S1.

SILAC analysis furthermore revealed a total of 35 serine and threonine phosphorylation sites on Atg9, which are all located in the cytosolic domains of the protein (Figure 2A; Figure S2A; Table S1). Six of these sites match the Atg1 consensus (Figure 2A; Figure S2B). Interestingly, phosphorylation of five out of the six sites matching the Atg1 kinase motif was absent or strongly reduced in Atg9 protein isolated from *atg1Δ* cells, showing that these sites are indeed phosphorylated in an Atg1-dependent manner in vivo (Figure 2B). The remaining predicted phosphorylation site, serine 657, could not be identified by mass spectrometry, most likely due to technical difficulties resulting from the arginine residue preceding serine 657.

To determine whether Atg1 directly phosphorylates Atg9 on additional serine residues, we synthesized 54 peptides spanning the Atg9 protein sequence and incubated them with soluble Atg1 kinase. The resulting phosphorylated peptides were then analyzed as in Figure 1D. Three out of 54 peptides spanning Atg9 were phosphorylated in vitro by Atg1 on a serine residue (Figure 2C). Two of these phosphorylation sites, serine 657 and 948, were among the six consensus motif sites. The lack of identification of the remaining four consensus sites is likely due to stochastic sampling of the mass spectrometry method used or to the poor flying nature of the peptides themselves, as their sequences were not chosen specifically for their ability to be detected but rather for their biological significance. Indeed, for several peptides, neither the naked nor the phosphorylated form was detected. We therefore cannot exclude the possibility that these peptides were phosphorylated in vitro but not detected by mass spectrometry. The third phosphorylated site at S602 also matched the Atg1 kinase consensus motif; however, it is located in a luminal exposed loop of Atg9 and is most likely not accessible to the Atg1 kinase in vivo. Taken together, the peptide approach shows that Atg1 selectively and directly phosphorylates at least two consensus sites in vitro.

To confirm these results, we performed additional in vitro phosphorylation experiments using as a substrate the C terminus of Atg9 (amino acids 757–997) purified from *Escherichia coli*. This fragment contains four of the six consensus phosphorylation sites (S802, S831, S948, and S969). Indeed, whereas soluble Atg1 complex purified from yeast readily phosphorylated the C-terminal Atg9^757–997^ fragment, phosphorylation was strongly decreased upon mutation of all four consensus phosphorylation sites (Figure 2D).

In summary, these findings suggest that Atg9 is a bona fide target of Atg1, which directly phosphorylates Atg9 on several sites in vitro and in vivo.

### Atg1 Phosphorylation of Atg9 Is Required for Cvt and Autophagy Function

**Figure 3:**
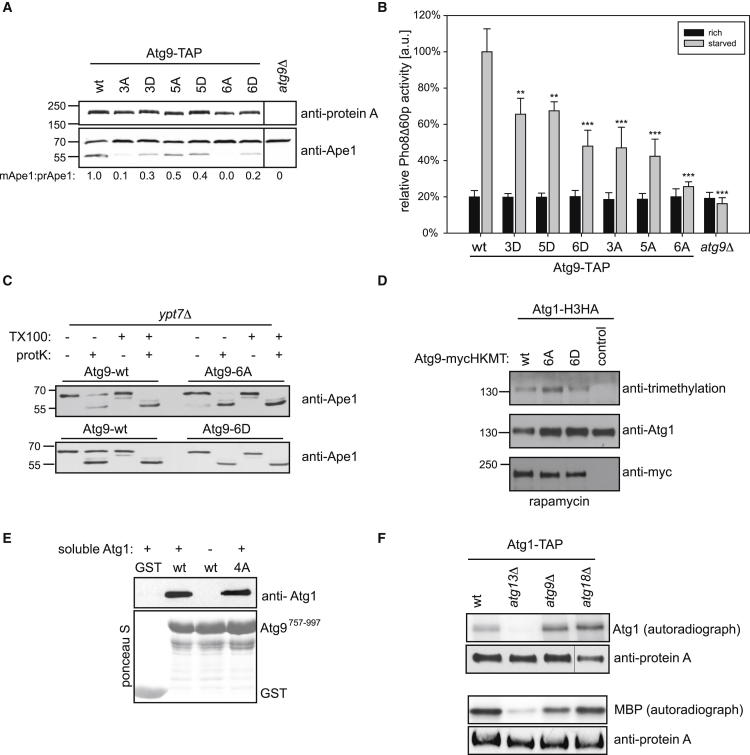
Atg1-Dependent Phosphorylation of Atg9 Is Required for Cvt and Autophagy Function (A) Endogenously TAP-tagged wild-type Atg9, Atg9 3A or 3D (S657A/D, S831A/E, S948A/D), 5A or 5D (S19A/D, S802A/D, S831A/E, S948A/D, S969A/D), 6A or 6D mutants (S19A/D, S657A/D, S802A/D, S831A/E, S948A/D, S969A/D), or *atg9Δ* cells were grown to mid-log phase. Processing of endogenous Ape1 was analyzed by western blotting and quantified by calculating the relative ratio of cleaved versus uncleaved Ape1 normalized to the wild-type. Expression of Atg9 proteins was assessed by anti-protein A western blotting. Note that the 5A/D mutants contain all the mutations as the 6A/D mutants except for S657, which is located close to a predicted transmembrane domain. (B) *pho8Δ60pho13Δatg9Δ* cells expressing TAP-tagged Atg9 wild-type, Atg9-3A, 3D, 5A, 5D, 6A, or 6D mutants, or an empty plasmid were grown to mid-log phase and starved for 5 hr in SD-N medium (starvation medium; 0.17% yeast nitrogen base without amino acids, 2% glucose). Pho8Δ60-specific alkaline phosphatase (ALP) activity (nmol/[min × mg]) was measured in four independent experiments as described in the Experimental Procedures, and the mean was plotted normalized to wild-type ALP activity. Differences from wild-type are statistically significant: ^∗∗^p < 0.01, ^∗∗∗^p < 0.001. Error bars represent normalized standard deviation. (C) *ypt7Δ* Atg9-myc wild-type or 6A or 6D mutant cells were treated with rapamycin and converted to spheroplasts. Total-cell extracts from lysed spheroplasts were centrifuged and the pellet fraction was either not treated or mixed with proteinase K (protK) and/or Triton X-100 (TX100). After protein precipitation, samples were analyzed by western blotting with anti-Ape1 antibodies. The *ypt7Δ* background was used to inhibit the fusion of autophagosomes with the vacuole to prevent Ape1 processing by vacuolar enzymes, which would complicate the comparison of autophagy mutants to wild-type cells. (D) Histone H3-HA (H3HA)-tagged Atg1 was expressed together with Atg9 wild-type or the Atg9-6A or Atg9-6D mutant fused to 9× myc and Suv39 methyltransferase (HKMT) in *atg1Δatg9Δ* cells, or as a control with Pbs2-HKMT in *atg9Δ* cells. Logarithmically growing cultures were treated with rapamycin for 1 hr, and methylation was assessed by western blotting with an anti-trimethylation-specific antibody after preparing cell extracts by freezer milling and HA immunoprecipitation of Atg1-H3HA. Note that HKMT-tagged Atg9 and H3HA-tagged Atg1 are fully functional. (E) GST, GST-Atg9^757–997^ wild-type, and the 4A mutant were expressed in *E. coli*, immobilized on beads, and incubated with soluble Atg1. Bound protein was assessed by anti-Atg1 western blotting, and the GST fusion proteins were visualized by Ponceau S staining. (F) Atg1-TAP, Atg1-TAP *atg9Δ*, Atg1-TAP *atg13Δ*, and Atg1-TAP *atg18Δ* cells were grown to mid-log phase and treated for 1 hr with rapamycin. Atg1 was immunoprecipitated and its autophosphorylation and model substrate (MBP) phosphorylation activity was measured by autoradiography in vitro. Anti-protein A blots shown in one panel are from the same blot with the same exposure. See also Figure S2.

To investigate whether these phosphorylation events are important for Atg9 function in vivo, we generated nonphosphorylatable and phospho-mimicking mutants of Atg9. All highly regulated serine residues were mutated to alanine (A) or aspartate/glutamate (D/E; S19A/D, S657A/D, S802A/D, S831A/E, S948A/D, S969A/D) and analyzed for their effect on the Cvt pathway or autophagy when expressed in *atg9Δ* cells. S657, S831, and S948 were analyzed individually, in all possible double-mutant permutations, as well as the triple mutant (3A, 3D; Figure S2C). In addition, all six sites were mutated simultaneously (6A, 6D) or lacking S657, which is located next to a transmembrane region (5A, 5D). All mutants were expressed at similar levels as the wild-type protein and cofractionated with membranes (Figure 3A; Figure S2D). First, we monitored Cvt activity by measuring Ape1 processing. Whereas the individual S-to-A mutations had little effect on the Cvt pathway (Figure S2C), Atg9 containing all six mutated serines (6A) near completely negated Cvt activity (Figure 3A). Various combinations of multiple S-to-A mutations behaved as intermediates between the two extremes. This suggests that phosphorylation of these residues is indeed required for Cvt activity, even though no single site is essential for Atg9 function. Equal expression levels, membrane association, and increasing severity of the mutant phenotype further support that the defect monitored is due to the lack of phosphorylation rather than misfolding of the mutant protein. Interestingly, the aspartate/glutamate phospho-mimicking mutations showed similar though less severe defects in Ape1 processing compared to the alanine mutations (Figure 3A), indicating that the mutations into charged residues are not sufficient to mimic the phosphorylated state of Atg9. Alternatively, cycles of phosphorylation and dephosphorylation may be required for Atg9 function in the Cvt pathway.

Next, we quantified bulk autophagy using the Pho8Δ60 assay that measures the autophagic delivery of the Pho8Δ60 phosphatase into the vacuole (Noda et al., 1995). As expected, Pho8Δ60 activity in wild-type cells was increased after 5 hr of nitrogen starvation, whereas no such increase was measured in *atg9Δ* cells. Interestingly, in Atg9 nonphosphorylatable mutant cells (3A, 5A, 6A), the starvation-induced increase in Pho8Δ60 activity amounted to only 10%–30% of that in wild-type cells, indicating that Atg9 phosphorylation is required for bulk autophagy in vivo (Figure 3B). Similarly, the phospho-mimicking mutants showed up to a 50% loss in autophagy activity. These results were confirmed by a GFP-Atg8 cleavage assay (Meiling-Wesse et al., 2002), in which processing of GFP-Atg8 was strongly inhibited in the Atg9 mutant cells (Figure S2E). In summary, these results demonstrate that Atg9 phosphorylation by Atg1 is required for the Cvt pathway and autophagy function in vivo.

### Atg9 Phosphorylation Is Essential for Autophagosome Formation

To determine whether the defect in autophagy observed in Atg9 mutant cells is due to a failure in early events such as the formation of the autophagosomal membrane or at a later stage of the process as the targeting to or fusion with the vacuole, we examined autophagosome closure using protease protection assays. Whereas in wild-type cells about 50% of Ape1 is protected from cleavage by proteinase K, indicating that Ape1 is present in autophagosomes, all was cleaved in the Atg9 mutants (Figure 3C; Figure S2F). These findings suggest that Atg9 phosphorylation is required at an early step of autophagy either to initiate autophagosome formation or to ensure the proper growth or closure of these double-membrane vesicles.

### Atg9 Interacts with Atg1 but Is Dispensable for Kinase Activity

As Atg1 phosphorylates Atg9 and both proteins are part of the PAS, these proteins are predicted to at least transiently interact. To test Atg1-Atg9 binding, we used an in vivo proximity assay based on fusing a histone lysine methyltransferase domain to a bait protein while fusing the substrate, histone H3, to the prey protein. Upon binding, the prey is stably methylated in vivo, which can subsequently be detected by immunoblotting using methylation-specific antibodies (Zuzuarregui et al., 2012). This assay revealed that Atg1 and Atg9 indeed interact in vivo, and that this interaction was not affected by the 6A or 6D mutations (Figure 3D). Similarly, soluble Atg1 was able to bind to the wild-type and nonphosphorylatable (4A) mutant of the recombinant Atg9 C-terminal fragment (Figure 3E). As expected, binding of Atg9 to Atg1 is not required for Atg1 kinase function in vivo, as Atg1 kinase purified from *atg9Δ* cells was found to be active (Figure 3F).

Together, these results show that Atg1 kinase activity and association occur independent of Atg9 phosphorylation and suggest that unphosphorylated Atg9 is a substrate of Atg1.

### Atg9 Phosphorylation Is Dispensable for Shuttling from and to the PAS

**Figure 4:**
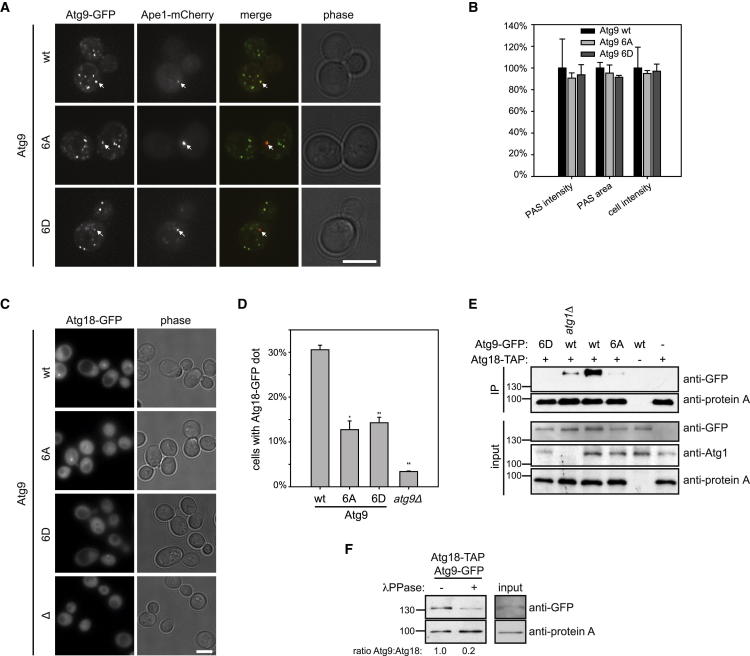
Atg9 Mutants Are Defective in PAS Recruitment of and Interaction with Atg18 (A) Localization of Atg9-GFP wild-type, Atg9-6A-GFP, and Atg9-6D-GFP was analyzed after 30 min of rapamycin treatment. PAS accumulation is marked by arrows. The scale bar represents 5 μm. (B) Mean PAS intensity, PAS area, and cell intensity were quantified. No statistically significant difference was found. Error bars represent standard deviation. (C and D) Dot formation of Atg18-GFP was quantified in *atg9Δ* cells containing Atg9-TAP wild-type, Atg9-6A-TAP, Atg9-6D-TAP, or an empty plasmid after a 1 hr rapamycin treatment. In two individual experiments at least 310 cells per mutant were counted blindly. The statistical significance of the difference from wild-type was calculated: P_6A_: 0.0148; P_6D_: 0.0094; P_delta_: 0.0014. ^∗^p < 0.05, ^∗∗^p < 0.01. Error bars represent standard deviation. Representative fluorescence images are shown in (C). The scale bar represents 5 μm. (E) *atg18Δ* Atg9-GFP wild-type, 6A or 6D, and *atg18Δatg1Δ* Atg9-GFP and *atg18Δatg9Δ* cells containing Atg18-TAP or an empty plasmid were grown to logarithmic phase and treated with rapamycin for 1 hr. Atg18 was immunoprecipitated, and its association with Atg9 was analyzed by anti-GFP immunoblotting. (F) *atg18Δ* Atg9-GFP cells containing Atg18-TAP were immunoprecipitated and incubated with or without λ-phosphatase. Bound protein was analyzed by anti-GFP western blotting. The total extracts are shown (input). See also Figures S3 and S4.

As Atg9 shuttling to and from the PAS depends on Atg1 (Reggiori et al., 2004), we examined whether Atg9 phosphorylation may regulate this process. As previously reported, both *atg1Δ* and kinase-dead Atg1-D211A mutant cells show accumulation of Atg9 at the PAS (Figure S3A) (Yamamoto et al., 2012), demonstrating that the kinase activity of Atg1 is indeed required for Atg9 dynamics. Furthermore, Atg1 contains an LC3-interaction region (LIR), which allows binding to Atg8/LC3 and thereby targets Atg1 to autophagosomes, where it might regulate autophagosome formation (Kraft et al., 2012). Mutation of the LIR motif in Atg1 (Atg1-V432A/E433A) results in normal PAS localization, but autophagosomal association is reduced (Kraft et al., 2012). Interestingly, this LIR mutant Atg1 did not alter Atg9 shuttling, implying that Atg1 regulates Atg9 retrieval specifically at the PAS rather than on autophagosomes or the vacuolar membrane. Mutation of all six phosphorylation sites on Atg9, which causes defective Cvt and autophagy, did not affect its PAS localization or its distribution between the PAS and the cytosol (Figures 4A and 4B). Also, the Atg9-6A phospho mutant accumulated at the PAS in *atg1Δ* cells similar to the Atg9 wild-type (Figure S3B), demonstrating that the mutations do not interfere with its PAS recruitment. Taken together, these results show that the phenotype of the Atg9 phospho mutants does not simply mimic that of an Atg1 deletion. Our findings suggest that Atg1-dependent phosphorylation of Atg9 does not affect its shuttling to and from the PAS, but regulates a different step in the autophagy pathway.

### Atg9 Phosphorylation Regulates Its Interaction with Atg18 and Atg18 Recruitment to the PAS

As both Atg1 and Atg9 are required to recruit the autophagy-essential Atg2 and Atg18 proteins to the PAS (Suzuki et al., 2007), we next analyzed whether their recruitment depends on Atg9 phosphorylation. As previously reported (Suzuki et al., 2007), both Atg2 and Atg18 formed a punctate structure representing PAS localization in wild-type cells upon rapamycin treatment. This PAS localization was lost in *atg9Δ* cells. Whereas Atg2 localized normally to the PAS in the Atg9 mutants (Figures S4A and S4B), Atg18 PAS localization was significantly decreased (Figures 4C and 4D). Also, Atg18 failed to localize to the PAS in the absence of active Atg1 (Figure S4C). These results demonstrate that Atg18 recruitment is to a large part dependent on Atg9 phosphorylation and suggest that the phospho-Atg9-independent Atg18 recruitment is not sufficient to rescue autophagy defects (compare Figures 3B and 4D).

In line with this notion, it has previously been reported that Atg9 interacts with Atg18 in an Atg1-dependent manner (Reggiori et al., 2004). We reasoned that the decreased recruitment of Atg18 to the PAS might be due to a loss of interaction with the Atg9 phosphorylation site mutants. Indeed, endogenously expressed Atg18-TAP (tandem affinity purification) was able to coprecipitate with wild-type Atg9-GFP, and this interaction was dependent on the presence of active Atg1 (Figure 4E; Figure S4D). Consistently, the interaction of Atg18 with Atg9 was decreased after phosphatase treatment (Figure 4F), and Atg18 binding to the Atg9-6A and Atg9-6D mutants was hardly detectable (Figure 4E). Taken together, our results show that Atg1-dependent phosphorylation of Atg9 is essential for its interaction with Atg18.

### Atg8 PAS Recruitment and Isolation Membrane Expansion Require Atg9 Phosphorylation

**Figure 5:**
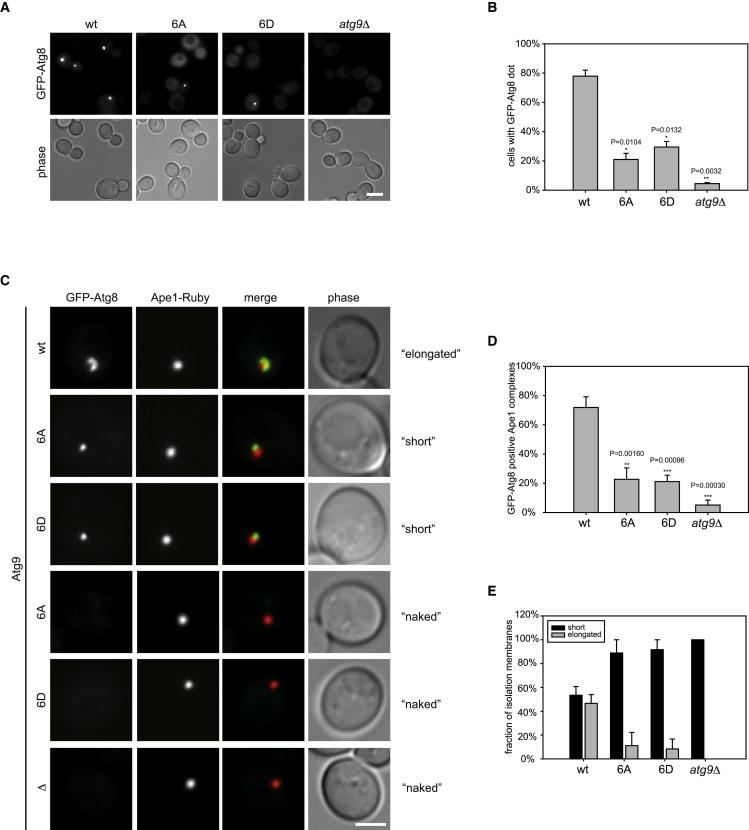
Atg9 Mutants Are Defective in Atg8 Recruitment to the PAS and Fail to Form an Isolation Membrane (A and B) Dot formation of Atg8-GFP was quantified in *atg9Δ* cells containing Atg9-TAP wild-type, Atg9-6A-TAP, Atg9-6D-TAP, or an empty plasmid after a 1 hr rapamycin treatment. In two individual experiments at least 285 cells per mutant were counted. Error bars represent standard deviation. Representative fluorescence images are shown in (A). The scale bar represents 5 μm. (C) GFP-Atg8 Ape1-mRuby2 Atg9-TAP wild-type, 6A, 6D, or *atg9Δ* strains containing CUP1-Ape1 were grown to logarithmic phase. Overexpression of Ape1 was induced by addition of 250 nM copper sulfate for 3 hr, and autophagy was induced by treating cells for 1 hr with rapamycin. The scale bar represents 2.5 μm. (D and E) The association of Atg8 with Ape1 structures (D) and the number of elongated isolation membranes (E) was quantified. Error bars represent standard deviation. See also Figure S4.

Atg8 localizes to the PAS during autophagosome formation. Phosphatidylethanolamine conjugation, the Atg12-Atg5-Atg16 complex, and Atg9 are all required for its proper recruitment (Kim et al., 2001; Suzuki et al., 2001). We therefore asked whether Atg1-dependent Atg9 phosphorylation is required for Atg8 recruitment to the PAS. As previously reported, Atg8 failed to be recruited to the PAS in *atg9Δ* cells (Figures 5A and 5B) (Suzuki et al., 2007). Similarly, the Atg9-6A and Atg9-6D mutants showed a strong defect in Atg8 puncta formation, strongly suggesting that Atg1-dependent phosphorylation of Atg9 is required for this recruitment process. This failure in recruitment was neither due to a defect in Atg5-Atg12 conjugation nor to a failure in the lipidation of Atg8, as both events took place in the Atg9-6A and Atg9-6D mutants to the same degree as in wild-type cells (Figures S4E and S4F). To test whether the small number of Atg8 dots in Atg9 mutant cells represents functional autophagosomes, we analyzed GFP-Atg8 structures in cells expressing giant Ape1 oligomers. Ape1 overexpression results in the formation of a giant Ape1 oligomer, which is too large to be fully enwrapped by the isolation membrane. These oligomers become partially enwrapped by Atg8-containing membranes in wild-type cells and are visible as cup-shaped structures by fluorescence microscopy, allowing the visualization and analysis of elongated isolation membrane intermediates (Suzuki et al., 2013). Only 20% of Atg9 mutant cells showed Atg8 recruitment to the giant Ape1 oligomers, whereas in 80% of wild-type cells Atg8 colocalized with Ape1, suggesting that Atg9 phosphorylation is required for this recruitment step (Figures 5C and 5D). As previously reported, wild-type cells formed elongated cup-shaped Atg8-positive structures, although Atg9 mutant cells only formed small patches (Figures 5C and 5E). Together, these findings show that Atg9 phosphorylation is required at a very early step of autophagosome formation to promote productive PAS assembly competent of isolation membrane formation.

**Figure 6:**
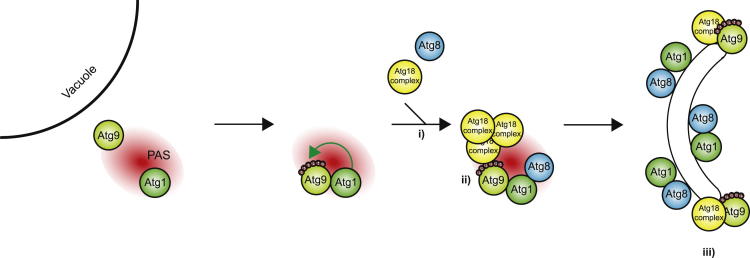
Atg1-Dependent Atg9 Phosphorylation Regulates Autophagy Atg9 vesicles are recruited to the PAS, where Atg9 is phosphorylated by Atg1 on six consensus sites. This allows the efficient recruitment of Atg18 and Atg8 to the PAS (i) and Atg9 binding to Atg18 (ii), which is required for isolation membrane elongation (iii) for autophagy and the Cvt pathway to function.

In summary, we have established a powerful screening method combining peptide arrays with quantitative mass spectrometry to identify kinase substrates. This method allowed us to determine the consensus phosphorylation sequence of the Atg1 kinase and the subsequent identification of 25 bona fide Atg1 kinase targets. Detailed analysis of one of these targets shows that (Figure 6, i) Atg9 phosphorylation by Atg1 facilitates efficient Atg8 and Atg18 recruitment to the PAS and is absolutely required for (Figure 6, ii) the interaction of Atg9 and Atg18 and (Figure 6, iii) isolation membrane expansion, which are necessary steps for autophagy and Cvt pathway function.

## Discussion

Atg1 kinase activity is essential for autophagy, but its mechanism of action is largely unknown. Here we determined the consensus sequence of the Atg1 kinase complex and used a genomic search to identify potential substrates. Among the candidates validated by in vitro phosphorylation, we identified the conserved core autophagy protein Atg9 as a direct Atg1 target. Atg9 phosphorylation by Atg1 is essential for its interaction with Atg18 (WIPI2 in mammals) and efficient recruitment of this key autophagy factor and Atg8 to the PAS. This in turn is required for PAS assembly capable of Atg8-positive isolation membrane formation and elongation.

Consensus motifs of a large number of yeast kinases have previously been analyzed using peptide arrays (Mok et al., 2010). However, these studies used overexpressed kinases, likely resulting in the loss of less abundant regulatory factors and possibly important secondary modifications on the kinase, both of which could influence substrate selectivity. Atg1 is a low-abundant protein in the cell, and its activity requires Atg13, another low-abundant protein. Therefore, we purified the native Atg1 kinase complex from yeast, preserving regulatory factors such as associated proteins and posttranslational modifications. Among other proteins, these isolated complexes contain the known regulators Atg13, Atg17, and Atg11, as verified by mass spectrometry. They show autophosphorylation activity as well as phosphorylation of myelin basic protein (MBP) as a model substrate, and both are increased approximately two-fold upon starvation or rapamycin treatment prior to complex isolation (Kijanska et al., 2010). Incubation of peptide arrays with native Atg1 kinase complexes revealed a selective motif distinct from known kinase target motifs, which tend to prefer either charged residues (protein kinase A and casein kinases) or proline (cyclin-dependent kinases and MAP kinases) (Mok et al., 2010; Pinna and Ruzzene, 1996). By contrast, Atg1 clearly preferred hydrophobic residues at multiple positions. Among previously characterized kinases, NimA-related kinases (yeast Kin3 and mammalian NEK6) also select hydrophobic residues at the −3 position, although they prefer aromatic over aliphatic residues (Lizcano et al., 2002; Mok et al., 2010). The notion of this Atg1 kinase consensus allowed us to identify a set of bona fide Atg1 kinase targets, among them the autophagy-essential protein Atg9.

Atg2 and Atg18 are essential for autophagy and the Cvt pathway. Atg18 contains a WD40 propeller and directly binds to Atg2 (Baskaran et al., 2012; Krick et al., 2012; Rieter et al., 2013), and both proteins require Atg9 for their proper PAS recruitment and function (Suzuki et al., 2007). Our results demonstrate that phosphorylation of Atg9 by Atg1 regulates its interaction with Atg18 and promotes Atg8 and Atg18 recruitment to the PAS and subsequent elongation of the isolation membrane, which is required for the Cvt pathway and autophagy to function (Figure 6). Interestingly, we show that when Atg1 cannot phosphorylate Atg9, Atg18 is still partially recruited to the PAS. However, this partial recruitment is apparently not sufficient to support autophagosome formation. Therefore, either the residual level of Atg18 recruitment lies under the threshold required at the PAS to promote these pathways, or it is the interaction of Atg9 with Atg18 rather than mere PAS recruitment that is essential for these pathways to function. As the latter is completely disrupted in the Atg9 mutants, the interaction of Atg9 with Atg18, possibly both in the cytoplasm and at the PAS, likely plays a central role in the promotion of autophagy. Additionally, Atg8 fails to be recruited efficiently to the PAS in the absence of Atg9 phosphorylation, and isolation membranes do not elongate, possibly due to the lack of Atg9-Atg18 interaction.

Atg9 phosphorylation by Atg1 is required both under rich conditions and upon starvation, as the Cvt pathway and autophagy are nonfunctional in Atg9 mutant cells. The serine-to-aspartate mutations in Atg9 seem unable to mimic the phosphorylated state. Although this unfortunately does not allow analysis of Atg9 function during continuous phosphorylation, it is conceivable that cycles of phosphorylation and dephosphorylation are required for progressive autophagy and the Cvt pathway. Further analyses of the precise mechanism, temporal control, and involvement of Atg9 phosphorylation during Atg9 vesicle cycling and PAS formation will be important directions for future studies.

Interestingly, although Atg1 kinase activity is required for Atg9 retrograde transport, direct phosphorylation of Atg9 by Atg1 was not required for this event, as fluorescence microscopy analyses revealed no significant alterations in the localization of nonphosphorylatable Atg9 mutants. Our data do not exclude the possibility that both antero- and retrograde trafficking kinetics are altered and the overall movement of Atg9 vesicles is slowed down. However, it seems more likely that Atg1 regulates Atg9 trafficking by other means than direct phosphorylation of Atg9. Thus, the critical Atg1 kinase substrate controlling Atg9 shuttling remains to be identified. Notably, the Atg1 LIR mutant, which is defective in binding to autophagosomes but still localizes to the PAS normally (Kijanska et al., 2010), shows no change in Atg9 trafficking behavior, implying that the function of Atg1 in regulating Atg9 transport might be restricted to the PAS.

With the complex interplay of many autophagy proteins in PAS function and autophagosome formation, future studies are required to unravel the precise interplay and mechanism underlying Atg1 signaling in autophagy and Cvt pathway function. The identification of Atg9 as an important and direct Atg1 kinase target adds another piece to this still largely unassembled puzzle.

## Experimental Procedures

Yeast strains and plasmids are listed in Tables S2 and S3.

### Purification of Soluble Atg1

Yeast cells were grown in 2 l of YPD medium to an OD_600_ of 2 and treated with 220 nM rapamycin for 1 hr, harvested by centrifugation, and washed in PBS, 2% glucose. Cells were then resuspended in a pellet volume of lysis buffer (PBS, 10% glycerol, 0.5% Tween-20, 1 mM NaF, 1 mM phenylmethylsulfonylfluoride, 1 mM Na_3_VO_4_, 20 mM β-glycerophosphate, protease inhibitor cocktail [Roche]) and frozen in droplets in liquid nitrogen. After cell disruption with a freezer mill (6770; SPEX), the extract was thawed in lysis buffer and cleared by centrifugation. The cleared extract was incubated with 160 μl of IgG-coupled magnetic beads (Dynabeads; Invitrogen) for 1 hr at 4°C with rotation. The beads were washed six times for 5–10 min in lysis buffer with rotation and cleaved with lysis buffer containing 0.5 mM DTT and TEV protease for 1 hr at 16°C with slow shaking.

### In Vivo Methylation-Interaction Assay

Atg1 was genomically tagged at the C terminus with histone H3-HA (H3HA), and Atg9 with a C-terminal histone lysine methyltransferase domain (HKMT) was cloned in a CEN plasmid under the control of its endogenous promoter. Both proteins were tested for functionality by analyzing Ape1 processing. The M-Track methylation assay was performed as described (Zuzuarregui et al., 2012). Methylation signals were enriched by immunoprecipitating Atg1-H3HA on HA agarose.

### Statistical Analysis

All p values were calculated using an unpaired two-tailed Student’s t test. A p value less than 0.05 was considered to be significant. The intensities from the MS1 filtering in Figure 2C were normalized to the values from the 60 min reactions, and the error bars represent the normalized standard deviation of the replicate injections. In Figure 3B, the mean and standard deviation were calculated from four individual experiments. P_3D_: 0.0025; P_5D_: 0.0020; P_6D_: 0.00022; P_3A_: 0.00033; P_5A_: 0.00013; P_6A_: 0.0000091; P_delta_: 0.0000006. Figures 4B, 4D, and 5B show the mean and standard deviation calculated from two individual experiments. Figures 5D and 5E show the mean and standard deviation calculated from four (wild-type) or two (mutant) individual experiments. For Figure 4B, at least 14 cells of each mutant were analyzed per experiment. For Figure 4D, at least 310 cells of each mutant per experiment were counted blindly. For Figure 5B, at least 280 cells of each mutant were counted per experiment. For Figures 5D and 5E, at least 25 cells of each mutant were analyzed per experiment.

Additional methods can be found in the Supplemental Experimental Procedures.

## Author Contributions

D.P., M.S., W.R., L.W., C.A.B., A.M., I.H., T.P., J.H.L., and C.K. performed the experiments shown in the manuscript. M.K., I.S., S.S.L., and A.B. performed initial experiments. D.P., W.R., L.W., C.A.B., A.M., B.E.T., R.A., G.A., M.P., and C.K. participated in the experimental design. M.P. and C.K. wrote the paper.

## Supplemental Information

Document S1. Figures S1–S5, Tables S2–S3, and Supplemental Experimental Procedures

Table S1. Detailed SILAC Data
