# Supplementary material for: Early Steps in Autophagy Depend on Direct Phosphorylation of Atg9 by the Atg1 Kinase
Source: Mol Cell. 2014 Feb 6;53(3):471–83. doi: 10.1016/j.molcel.2013.12.011 (PMC3978657; doi:10.1016/j.molcel.2013.12.011)
Supplement: Document S1. Figures S1–S5, Tables S2–S3, and Supplemental Experimental Procedures [file mmc1.pdf]

Molecular Cell, Volume *53*

Supplemental Information

Early Steps in Autophagy Depend on Direct Phosphorylation of Atg9 by the Atg1 Kinase

Daniel Papinski, Martina Schuschnig, Wolfgang Reiter, Larissa Wilhelm, Christopher A. Barnes, Alessio Majolica, Isabella Hansmann, Thaddaeus Pfaffenwimmer, Monika Kijanska, Ingrid Stoffel, Sung Sik Lee, Andrea Brezovich, Jane Hua Lou, Benjamin E. Turk, Ruedi Aebersold, Gustav Ammerer, Matthias Peter, and Claudine Kraft

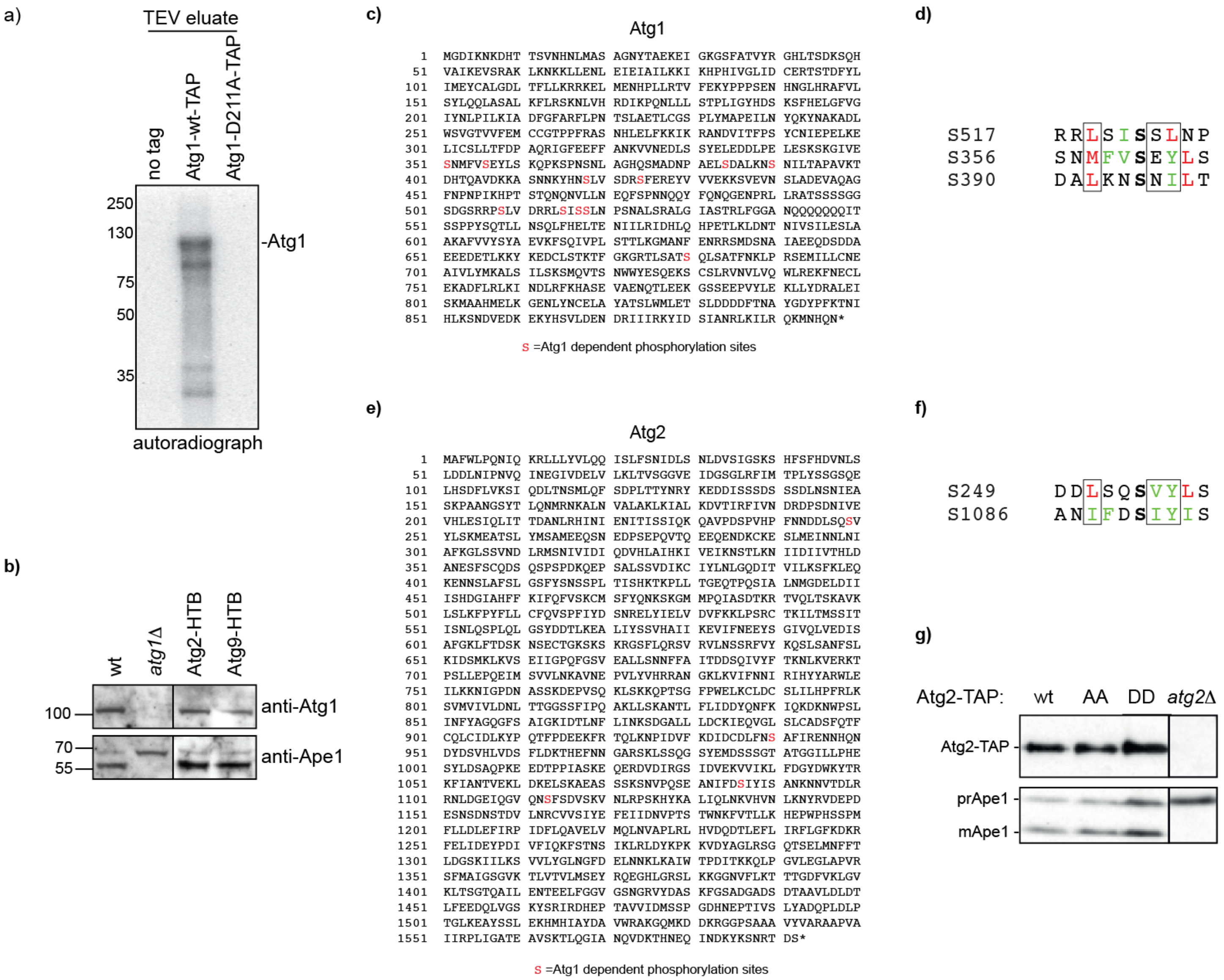

Figure S1, related to Figure 1

(a) The entire autoradiograph of Figure 1b is shown. (b) Wild-type, *atg1Δ* and genomically tagged Atg2-HTB and Atg9-HTB cells were grown to mid log phase. Cell extracts were prepared by TCA and analysed by anti-Atg1 and anti-Ape1 Western blotting. Blots shown in one panel are from the same blot with the same exposure. (c) *atg1Δ* strains containing Atg1-HTB and Atg1-D211A-HTB yeast cells were grown to mid log phase and treated with rapamycin for 45 minutes. Atg1 was affinity purified and subjected to quantitative mass spectrometric phosphorylation mapping by SILAC (13C/heavy wild-type vs. 12C/light D211A). Atg1 dependent sites are indicated in red (d) Alignment of Atg1's auto-phosphorylation sites S517, S356 and S390 as described in Fig. 1f. (e) Wild-type and *atg1Δ y*east cells containing endogenously tagged Atg2-HTBeaq were grown to mid log phase and rapamycin treated for 45 minutes. Atg2 was affinity purified and subjected to quantitative mass spectrometric phosphorylation mapping by SILAC (13C/heavy wild-type vs. 12C/light *atg1Δ*). Atg1 dependent sites are indicated in red. (f) Alignment of Atg2's phosphorylation sites S249 and S1086 as described in Fig. 1f. (g) Atg9-GFP *atg2Δ* yeast cells containing Atg2-TAP wild-type, S249A S1086A or S249D S1086D double mutants or empty plasmid were grown to mid log phase. Cell extracts were prepared by TCA and analysed by anti-Ape1 Western blotting. Blots shown in one panel are from the same blot with the same exposure.

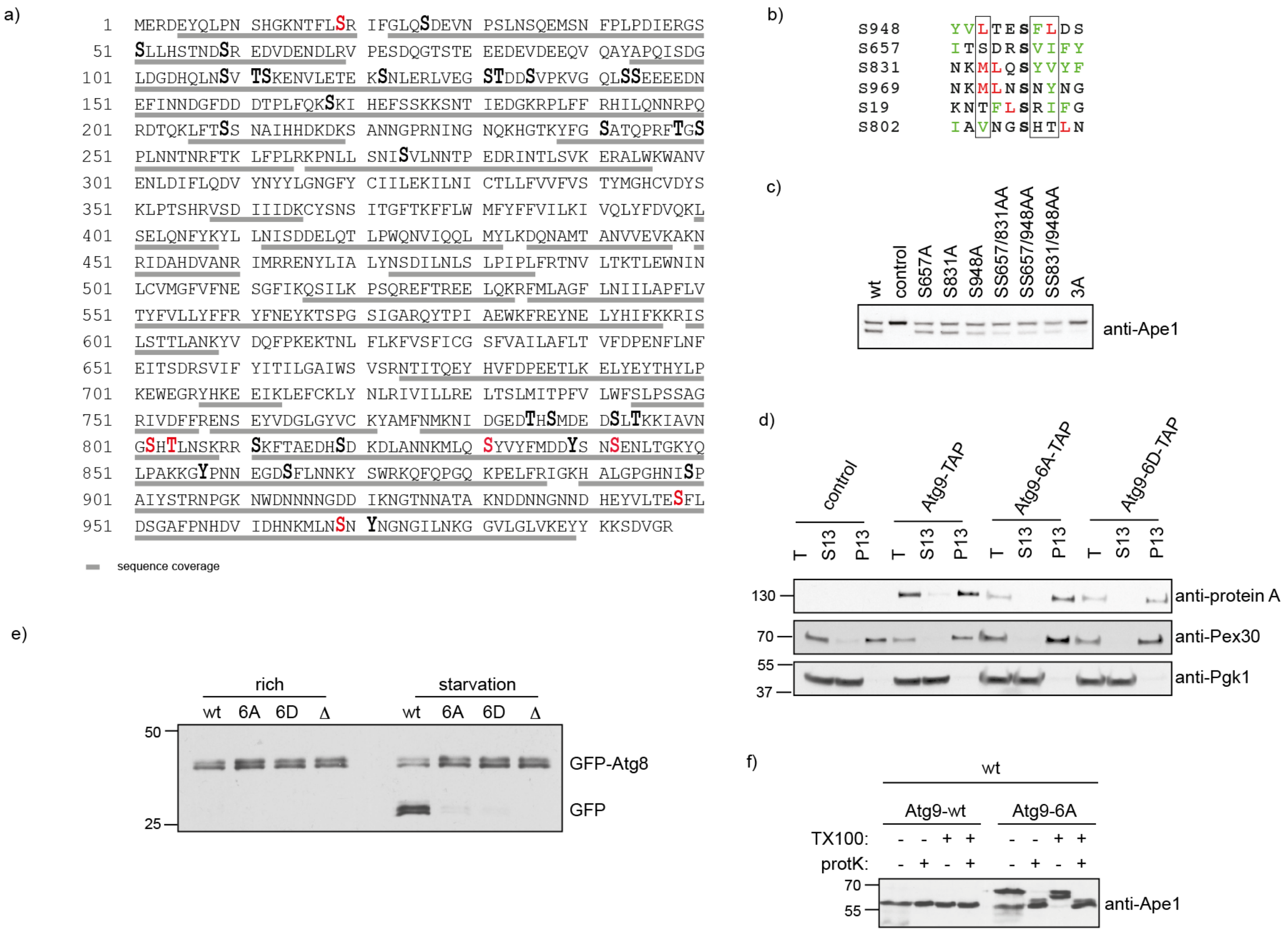

Figure S2, related to Figure 2 and 3

(a) Sequence coverage of Fig. 2a. (b) Alignment of the six phosphorylation sites on Atg9 as described in Fig. 1f. (c) GFP-tagged wild-type (wt) Atg9, the Atg9 3A (S657A, S831A, S948A) and the single and double mutants thereof expressed from a centromeric plasmid in *atg9Δ* cells were grown to mid log phase. Processing of endogenous Ape1 was analysed by Western blotting. (d) Exponentially growing wild-type (control), Atg9-TAP, Atg9-6A-TAP or Atg9-6D-TAP cells were treated for 2h with rapamycin. Cells were then lysed and the extract (T) separated into a cytoplasmic (S13) and a 13'000x g membrane (P13) fraction. The fractions were analysed by Western blotting with anti-PAP (Atg9), anti-Pgk1 (cytoplasmic control) and anti-Pex30 (membrane control) antibodies. (e) Atg9-TAP wild-type, 6A, 6D mutant or *atg9Δ* cells containing GFP-Atg8 were starved for 4 hours. TCA samples were collected and subjected to anti-GFP Western blotting. (f) Atg9-myc wild-type or the 6A mutant cells were treated with rapamycin and converted to spheroplasts. Total cell extracts from lysed spheroplasts were centrifuged and the pellet fraction was either not treated, or mixed with proteinase K (protK) and/or Triton X-100 (TX100). After protein precipitation, samples were analyzed by Western blotting with anti-Ape1 antibodies. Note that in Fig. 3c the same experiment is shown in a *ypt7Δ* background. *ypt7Δ* abolishes the fusion of autophagosomes with the vacuole to prevent Ape1 processing by vacuolar enzymes, allowing an easier comparison of autophagy mutants to wild-type cells.

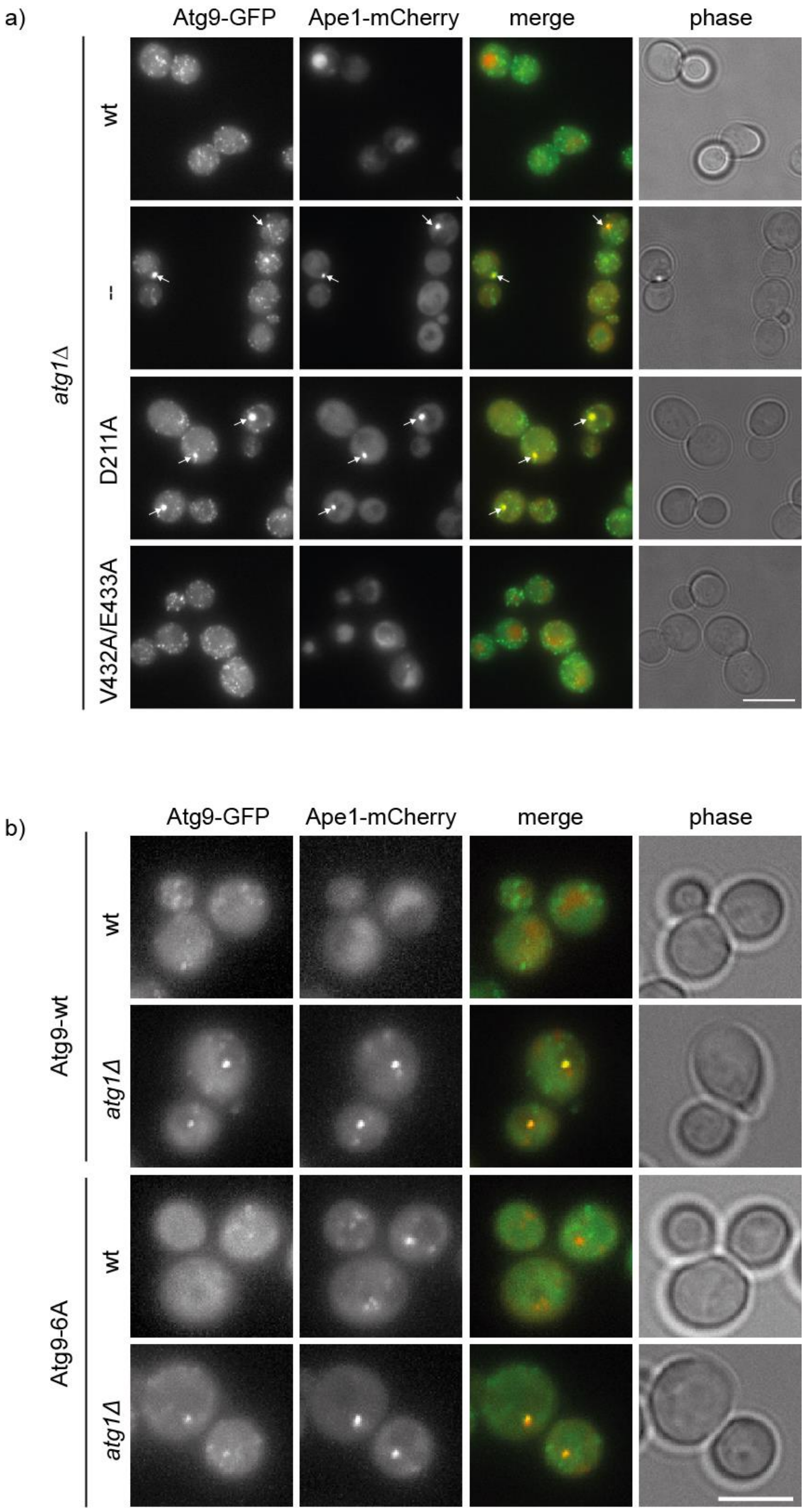

Figure S3, related to Figure 4

(a) Trafficking of Atg9-GFP was analyzed in wild-type, *atg1Δ*, Atg1-D211A and Atg1-V432A/E433A cells after starvation. Cells contained Ape1-mCherry to mark the PAS. PAS accumulation is marked with an arrow. Scale bar: 7.5 µm. (b) Trafficking of Atg9-GFP wild-type and the 6A mutant was analysed in wild-type and *atg1Δ* cells after 30 minutes of rapamycin treatment. Cells contained Ape1-mCherry to mark the PAS. Scale bar: 5 µm.

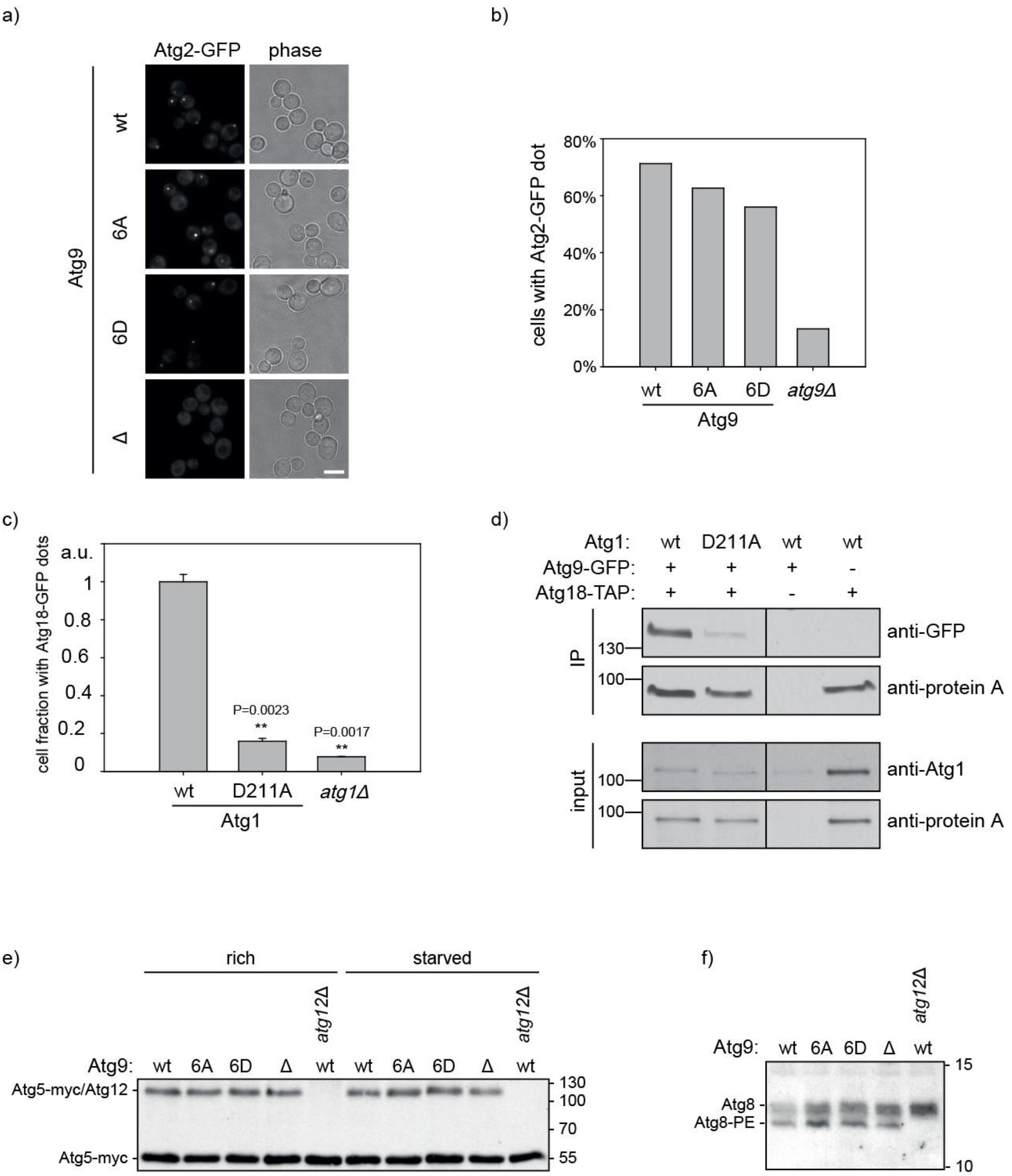

Figure S4, related to Figure 4 and 5

(a,b) Dot formation of Atg2-GFP was quantified as described in Fig. 4c,d. Scale bar: 5 μm. (c) Atg18-GFP *atg1Δ* cells containing Atg1 wild-type, Atg1-D211A or *atg1Δ* were analyzed for puncta formation as described in Fig. 4d. All values have been normalized to wild-type. Error bars: standard deviation. (d) Atg9-GFP *atg18Δ atg1Δ* or *atg18Δ atg9Δ* cells containing Atg18-TAP or an empty plasmid and Atg1 wild-type or Atg1-D211A were grown to logarithmic phase and treated with rapamycin for 1h. Atg18 was immunoprecipitated and its association with Atg9 was analyzed by anti-GFP immunoblotting. Panels shown are from the same blot with the same exposure. (e) Endogenously tagged Atg9-TAP wild-type (wt), 6A, 6D, *atg9Δ* or *atg12Δ* cells containing Atg5-myc were grown to log phase or starved for 4h and samples were prepared by TCA precipitation. Conjugation of Atg5 to Atg12 was monitored by anti-myc Western blotting. (f) Endogenously tagged Atg9-TAP wild-type (wt), 6A, 6D, *atg9Δ* or *atg12Δ* cells were grown to log phase and starved for 4h, and samples were prepared by TCA precipitation. Lipidation of Atg8 was analyzed on a 15% SDS-PAGE containing 6M urea by anti-Atg8 Western blotting.

Figure S5, related to Figure 1 and 2

In vitro phosphorylation reactions were performed using immunopurified soluble active Atg1 against synthesized candidate peptides. Reactions were sampled at 5 minutes, 30 minutes, and 60 minutes. Each time point was injected twice into the LC-MS/MS system and label-free quantification was performed on the precursor ion chromatographic traces that correlated to the identified phosphorylated peptide for each injection. Here, the masses for M, M + 1, and M + 2 are shown which represent the naturally occurring isotopic distribution of the precursors. The isotopic dot product score represents the expected distribution of isotopes (0 to 1 where 1 is the highest).

Table S2: Yeast strains used in this study.

Name	Genotype	Background	Source
BY4741	Mat a; his3Δ1, leu2Δ0, met15Δ0, ura3Δ0	BY474x	Euroscarf
BY4743	Diploid; his3Δ1/ his3Δ1, leu2Δ0/ leu2Δ0, met15Δ0/ MET15, ura3Δ0/ ura3Δ0, lys2D0/ LYS2	BY474x	Euroscarf
BY SILAC	BY4741; lys1::KanMX6, arg4::KanMX6, lys2D0 or LYS2	BY SILAC	(Gruhler et al., 2005)
WR636	Mat a; Atg9-HTBeaq:NatMX6	BY SILAC	this study
IH59	Mat a; Atg9-HTBeaq:NatMX6, atg1::HisMX6	BY SILAC	this study
yCK819	Mat a; Atg2-HTBeaq:NatMX6	BY SILAC	this study
IH178	Mat a; Atg2-HTBeaq:NatMX6, atg1::HisMX6	BY SILAC	this study
WR 937	Atg1-HTBeaq:NatMX6	BY SILAC	this study
WR 938	Atg1 ^{D211A}-HTB:NatMX6	BY SILAC	this study
yCK414	Mat a; atg9::kanMX6	BY474x	this study
yCK415	Mat alpha; atg9::kanMX6, MET15, lys2D0	BY474x	this study
yCK660	Mat a; atg1::KanMX6	BY474x	(Kijanska et al., 2010)
yMS12	Mat a; Atg1-TAP:HisMX6	BY474x	this study
yMS13	Mat a; Atg1 ^{D211A}-TAP:HisMX6	BY474x	this study
yMS25	Mat a; ATG9-TAP:HisMX6	BY474x	this study
yMS26	Mat a; atg9 $^{3A: S657A\ S831A\ S948A}$ -TAP:HisMX6	BY474x	this study
yMS27	Mat a; atg9 $^{3D: S657D\ S831E\ S948D}$ -TAP:HisMX6	BY474x	this study
yMS28	Mat a; atg9 $^{5A: S19A\ S802A\ S831A\ S948A\ S969A}$ -TAP:HisMX6	BY474x	this study
yMS29	Mat a; atg9 $^{5D: S19D\ S802D\ S831E\ S948D\ S969D}$ -TAP:HisMX6	BY474x	this study
yMS30	Mat a; atg9 $^{6A: S19A\ S657A\ S802A\ S831A\ S948A\ S969A}$ -TAP:HisMX6	BY474x	this study
yMS31	Mat a; atg9 $^{6D: S19D\ S657D\ S802D\ S831E\ S948D\ S969D}$ -TAP:HisMX6	BY474x	this study
yMS18	Mat a; Atg9-9xmyc:Ura	BY474x	this study
yMS23	Mat a; atg9 $^{6A: S19A\ S657A\ S802A\ S831A\ S948A\ S969A}$ -9xmyc:Ura	BY474x	this study
yMS52	Mat a; Atg9-9xmyc:Ura, ypt7::NatMX6	BY474x	this study
yMS54	Mat a; atg9 $^{6A: S19A\ S657A\ S802A\ S831A\ S948A\ S969A}$ -9xmyc:Ura, ypt7::NatMX6	BY474x	this study
yMS56	Mat a; atg9 $^{6D: S19D\ S657D\ S802D\ S831E\ S948D\ S969D}$ -9xmyc:Ura, ypt7::NatMX6	BY474x	this study
yDP15	Mat a; atg9::natMX6, pho8::pho8Δ60:HisMX6, pho13::kanMX6, MET15	BY474x	this study
yLW22	Mat alpha; ATG1-H3HA:URA3, atg9::KanMX6	BY474x	this study
yCK5	Mat a; ATG1-TAP:HisMX6	BY474x	Euroscarf
yCK717	Mat a; ATG1-TAP:His MX6, atg9::KanMX6, MET15	BY474x	this study
yCK489	Mat alpha; ATG1-TAP:HisMX6, atg13::KanMX6	BY474x	(Kraft et al., 2012)
yCK748	Mat a; atg12::KanMX6	BY474x	this study

yMS37	Mat alpha; ATG1-TAP:HisMX6, atg18::KanMX6	BY474x	this study
yDP85	Mat a; ATG9-GFP:HisMX6, APE1-mCherry	BY474x	this study
yDP95	Mat a; atg9 6A: S19A S657A S802A S831A S948A S969A -GFP:HisMX6, APE1-mCherry, MET15	BY474x	this study
yDP99	Mat a; atg9 6D: S19D S657D S802D S831E S948D S969D -GFP:HisMX6 APE1-mCherry, MET15, lys2D0	BY474x	this study
yDP83	Mat alpha; ATG18-GFP:HisMX6, atg9::KanMX6, MET15	BY474x	this study
yDP141	Mat a; ATG9-GFP:HisMX6, APE1-mCherry, atg1::KanMX6	BY474x	this study
yDP35	Mat a; ATG9-GFP:HisMX6, atg18::KanMX6	BY474x	this study
yDP37	Mat a; atg9 3A: S657A, S831A, S948A -GFP:HisMX6, atg18::KanMX6	BY474x	this study
yDP39	Mat alpha; atg9 3D: S657D, S831E, S948D -GFP:HisMX6, atg18::KanMX6, MET15, lys2D0	BY474x	this study
yDP41	Mat a; atg9::KanMX6, atg18::KanMX6	BY474x	this study
yDP112	Mat a; atg1::KanMX6, atg9 6A: S19A S657A S802A S831A S948A S969A -GFP:HisMX6, APE1-mCherry, MET15	BY474x	this study
yDP293	Mat alpha; ATG9-TAP:HisMX6, Ape1-mRuby:KanMX6, sfGFP-Atg8	BY474x	this study
yDP299	Mat alpha; atg9 6A: S19A S657A S802A S831A S948A S969A -TAP:HisMX6, Ape1-mRuby:KanMX6, sfGFP-Atg8	BY474x	this study
yDP306	Mat alpha; atg9 6D: S19D S657D S802D S831E S948D S969D -TAP:HisMX6, Ape1-mRuby:KanMX6, sfGFP-Atg8	BY474x	this study
yDP31	Mat a; Atg9-GFP:HisMX6, atg2::KanMX6	BY474x	this study
yMK181	Mat alpha; Atg2-GFP:HisMX6, atg9::KanMX6, MET15	BY474x	this study
yDP124	Mat alpha; Atg2-GFP:HisMX6, Atg9-9xmyc:UraMX6, MET15	BY474x	this study
yDP129	Mat alpha; Atg2-GFP:HisMX6 atg9 6A: S19A S657A S802A S831A S948A S969A -9xmyc:UraMX6, MET15	BY474x	this study
yDP130	Mat alpha; Atg2-GFP:HisMX6 atg9 6D: S19D S657D S802D S831E S948D S969D -9xmyc:UraMX6, MET15	BY474x	this study
yDP148	Mat a; atg1::KanMX6, Atg18-GFP:HisMX6	BY474x	this study
yDP345	Mat a; atg9::NatMX6, Ape1-mRuby:KanMX6, sfGFP-Atg8	BY474x	this study
yDP44	Mat a; Atg9-GFP:HisMX6, atg1::NatMX6, atg18::KanMX6	BY474x	this study

All strains of *S. cerevisiae* S288C BY474x genetic background are derived from the diploid strain BY4743 and carry the following markers: his3Δ1; leu2Δ0; met15Δ0; ura3Δ0 except if stated otherwise.

Table S3: Plasmids used in this study.

Name	Characteristics	Source
pRS315, pRS415	CEN LEU2	(Sikorski and Hieter, 1989)
pRS316, pRS416	CEN URA3	(Sikorski and Hieter, 1989)
pCK694	pRS415: Atg9-TAP	this study
pCK590	pRS415: Atg9 $^{3A:\ S657A,\ S831A,\ S948A}$-TAP	this study
pCK588	pRS415: Atg9 $^{3D:\ S657D,\ S831E,\ S948D}$ -TAP	this study
pDP59	pRS415: Atg9 $^{5A:\ S19A\ S802A\ S831A\ S948A\ S969A}$ -TAP	this study
pDP60	pRS415: Atg9 $^{5D:\ S19D\ S802D\ S831E\ S948D\ S969D}$ -TAP	this study
pDP57	pRS415: Atg9 $^{6A:\ S19A\ S657A\ S802A\ S831A\ S948A\ S969A}$ -TAP	this study
pDP58	pRS415: Atg9 $^{6D:\ S19D\ S657D\ S802D\ S831E\ S948D\ S969D}$ -TAP	this study
pLW49.1	pRS415: Atg9-mycHKMT	this study
pLW55	pRS415: Atg9 $^{6A:\ S19A\ S657A\ S802A\ S831A\ S948A\ S969A}$ -mycHKMT	this study
pLW56	pRS415: Atg9 $^{6D:\ S19D\ S657D\ S802D\ S831E\ S948D\ S969D}$ -mycHKMT	this study
IC60	YCp111: Pbs2-HKMT	(Kraft et al., 2012)
SMC270	pRS316: Atg5-myc	(Romanov et al., 2012)
SMC281	pGEX4T1: GST-Atg9 $^{757\text{-}997}$	this study
pCK711	pGEX4T1: GST-Atg9 $^{757\text{-}997,\ 4A:\ S802A\ S831A\ S948A\ S969A}$	this study
pCK352	pRS315: Atg1 $^{V432A\ E433A}$	(Kraft et al., 2012)
pCK178	pRS315: Atg1	(Kijanska et al., 2010)
pCK581	pRS315: Atg1 ^{D211A}	this study
pCK320	pRS315: Atg1-TAP	(Kraft et al., 2012)
pCK371	pRS315: Atg18-TAP	this study
pCK782	pRS315:promCUP1-Ape1	this study
pCK364	pRS315: Atg2-TAP	this study
pCK574	pRS315: Atg2 $^{S249A\ S1086A}$-TAP	this study
pCK562	pRS315: Atg2 $^{S249D\ S1086D}$ -TAP	this study
pCK48	pRS315: GFP-Atg8	(Kraft et al., 2012)
pDP1	pRS316: Atg1	this study
pCK633	pRS316: Atg1 ^{D211A}	this study

Table S4: Peptides analysed in Figure 2c.

Peptides were synthesized to cover the entire sequence of Atg9 such that each of the potentially phosphorylated residues was flanked by at least 4 amino acids on the N- or C-terminal ends.

Synthesized Atg9 Peptide Sequences	Naked Peptide Identified in Separate MS Injections of Peptide Library	Synthesized Atg9 Peptide Sequences	Naked Peptide Identified in Separate MS Injections of Peptide Library
NIDGEDTHSMDEDSLTK	yes	IFGLQSDEVNPSLNSR	yes
LVEGSTDDSVPK	yes	YFNEYKTSPGSIGAR	no
MLQSYVYFMDDYSNSENLTGK	yes	VIFYITILGAIWSVSR	no
YVDQFPKEKTNLFLK	yes	SLNSQEMSNFPLPDIER	yes
VDYSKLPTSHRVSDIIIDK	yes	PFVLWFSLPSSAGR	yes
TSPGSIGARQYTPIAEWK	yes	NTNRFTKLFPLR	no
SSKKSNTIEDGK	no	KPNLLSNISVLNNTPEDR	yes
PEETLKELYEYTHYLPK	yes	IILAPFLVTYFVLLYFFR	no
PEDRINTLSVKERALWK	no	GNQKHGTKYFGSATQPR	yes
NTITQEYHVFDPEETLK	yes	ENVLETEKSNLER	yes
NNDGFDDDTPLFQK	yes	DKDKSANNGPR	no
IIIDKCYSNSITGFTK	yes	YLLNISDDELQTLPWQR	yes
IAVNGSHTLNSK	yes	NNEGDSFLNNKYSWRKQFQR	no
HIFKKRISLSTTLANK	yes	ILAFLTVFDPENR	yes
GYPNNEGDSFLNNK	yes	DHEYVLTESFLDSGAFPNR	yes
GFTKFFLWMFYFFVILK	no	SKFTAEDHSDKDLR	yes
FVFNESGFIKEFTREELQK	yes	LIALYNSDILNLSLPIPLR	yes
MLQSYVYFMDDYSNSENLTGK	no	LFLKFVSFICGSFVAILR	no
FFRENSEYVDGLGYVCK	yes	NTFLSRIFGLR	yes
DVQKLSELQNFYK	yes	GDHQLNSVTSKENVLR	yes
DQNAMTANVVEVK	yes	APQISDGLDGDHQLR	yes
MLNSNYNGNGILNK	yes	HALGPGHNISPAIYSTR	yes
DEYQLPNSHGK	yes	PLFQKSKIHEFSSKKSNTIR	yes
DTQKLFTSSNAIHHDK	yes	LPIPLFRTNVLTKTLEWNIR	yes
FLNFEITSDRSVIFYR	yes	ILNICTLLFVVFVSTYMGR	no
IVILLRELTSLMITPFVR	yes	IKNGTNNATAKNDDNNGR	no
DIERGSSLLHSTNDSREDVR	yes	VPESDQGTSTEEER	yes
FTGSPLNNTNR	yes	VGQLSSEEEEDNER	yes

Supplemental experimental procedures

Yeast strains, growth conditions and antibodies. Yeast strains are listed in Supplementary Table S2. Genomic mutations of *ATG1* and *ATG9* were generated by homologous recombination of the mutant protein into the respective deletion strain. Yeast cells were grown in synthetic medium (SD: 0.17% yeast nitrogen base, 0.5% ammonium sulfate, 2% glucose, amino acids as required) or rich medium (YPD; 1% yeast extract, 2% peptone, 2% glucose). For starvation induction, cells in early logarithmic phase were washed and resuspended in starvation medium (SD-N: 0.17% yeast nitrogen base without amino acids, 2% glucose) for 2-4h, unless stated otherwise. Rapamycin was used at a final concentration of 220 nM.

The following antibodies were used in this study: mouse monoclonal anti-GFP antibody (Roche), rabbit polyclonal PAP antibody (Sigma), rabbit polyclonal anti-TAP antibody (Open Biosystems), mouse monoclonal anti-GST antibody (Sigma), mouse monoclonal anti-Pgk1 antibody (Invitrogen), mouse monoclonal anti-HA antibody (Roche), mouse anti-trimethylation specific antibody (Zuzuarregui et al., 2012). Anti-Atg1 and anti-Atg13 antibodies were kindly provided by Daniel Klionsky, University of Michigan. Anti-Pex30 antibody was a kind gift of Cecile Brocard. Polyclonal anti-Ape1 antibody was generated by immunizing rabbits with a synthetic peptide corresponding to amino acids 168-182.

Plasmid construction. Plasmids are listed in Supplementary Table S3. Site-directed mutagenesis was performed by QuikChange mutagenesis (Stratagene), and verified by sequencing. *ATG9* with its endogenous promoter (547 bases) was amplified from genomic DNA and ligated into the pRS416 and pRS415 vectors using BamHI and PstI restriction sites. *ATG18* with its endogenous promoter (650 bases) was amplified from genomic DNA and ligated via NotI-SbfI digested PCR into NotI-PstI cut pRS315. Fusion proteins of Atg9 and Atg18 were generated by ligating PCR-amplified TAP, GFP, 3xH3-3xHA and 3xmyc-HKMT (histone lysine methyltransferase SUV39H1 containing the H320R mutation (Zuzuarregui et al., 2012)) tags via PstI or Sbf1 and SalI to the C-terminus of the respective protein. pGEX4T1-Atg9$^{757-997}$ wild-type and 4A mutant were generated by PCR amplification of the specific region and ligation via BamHI and SmaI. The Cup1-Ape1 plasmid was generated by ligating the PCR amplified Cup1 promoter

via SalI and HindIII into pRS315 and subsequently adding the PCR amplified Ape1 ORF and its terminator via HindIII and NotI.

Yeast cell extract preparation, immunoprecipitations, TEV cleavage and the Pho8Δ60 assay. Protein extraction with TCA and immunoblotting was carried out as described previously (Kijanska et al., 2010). To solubilize Atg9, TCA samples were beaten with glass beads after resuspension in urea loading buffer (116mM TrisHCl pH 6.8, 4.9% glycerol, 7.99 M urea, 143 mM β-mercaptoethanol, 8% SDS). To prepare extract under non-denaturing conditions, cells were grown in rich or selective medium to early logarithmic phase and harvested by centrifugation. Cell extracts were prepared by freezer milling (Kraft et al., 2012) (Fig. 1,3,4) , or by glass bead lysis (Fig. 4d).

HA and proteinA immunoprecipitations were carried out as described previously (Kijanska et al., 2010; Kraft et al., 2012). For GFP immunoprecipitations, GFP-Trap agarose (ChromoTek) was used. For TEV cleavage, bound protein was incubated with TEV protease in a total of two bead volumes lysis buffer containing 0.5 mM DTT for 1 h at 16ºC.

The Pho8Δ60 assay was performed as described (Klionsky, 2007). To ensure the linear range of the enzymatic reaction, the assay was performed using two different incubation times.

Purification of GST-Atg9 C-terminal fragments. Amino acids 757-997 of Atg9 of either wild-type or the 4A mutant were cloned into pGEX4T1 resulting in N-terminal GST fusion proteins. Proteins were purified from *E.coli* Rosetta pLySS cells. Cells were grown at 37ºC to an OD600 of 0.8, induced with 1mM IPTG and grown for a further 5 hours. Cells were harvested and resuspended in extraction buffer (20 mM Tris HCl pH 7.5, 250 mM NaCl, 0.5 mM EDTA, 1mM PMSF, 0.05% TX-100, 100 µg/ml DNase I (Sigma), protease inhibitor cocktail (Roche)). Cells were lysed by freeze-thawing and sonication, and supernatants prepared by 10'000 x g centrifugation. The supernatant was incubated with glutathione-beads (GE Healthcare) for 1 hour. Beads were washed extensively with wash buffer (10 mM NaPi pH 7.4, 250 mM NaCl, 0.5 mM EDTA, 1mM PMSF, 0.05% TX-100, protease inhibitor cocktail (Roche)) and bound protein was analyzed by Coomassie or Ponceau staining.

Cell fractionation and proteinase protection assay. Yeast cells treated with 220nM rapamycin for 2 hours were washed and incubated with 0.2 ml/OD DTT buffer (10mM DTT, 10mM Tris pH 9.4; 15 min, 30°C), washed and resuspended in 25 OD/ml SP buffer (1M sorbitol, 20 mM PIPES pH 6.8) and spheroplasted by 0.4mg/ml Zymolyase-20T (MP Biomedicals) treatment for 45 min at 30ºC. Spheroplasts were collected by centrifugation (2000g for 5 min), washed in SP buffer and lysed with osmotic lysis buffer (200 mM sorbitol, 20 mM PIPES pH 6.8, 5mM $MgCl_2$, "input") to preserve organelle-enclosed particles. After three 500g centrifugation steps to remove unbroken cells, the resulting total lysate ("T") was further separated into a 13,000 x g supernatant ("S") and a pellet fraction ("P"). The efficiency of fractionation and distribution of Atg9 was determined by immunoblot analysis using anti-PAP, anti-Pgk1, and anti-Pex30 antibodies. For proteinase protection assays, the 13,000 x g pellet was resuspended in osmotic lysis buffer with or without proteinase K (50 µg/ml, AppliChem) and 0.2% Triton X-100. After 30 min incubation on ice, samples were precipitated with TCA and analyzed by Western blotting with anti-Ape1 antibodies.

Kinase assays. Kinase assays were performed as described (Kraft et al., 2012). For *in vitro* phosphorylation of $Atg9^{757-997}$, GST-tagged protein was immunoprecipitated and incubated with the phosphorylation mixture containing 1 µl of soluble Atg1 kinase. After 20 minutes incubation, the $Atg9^{757-997}$ bound beads were washed three times to remove soluble Atg1 and radioactivity incorporation was assessed by phosphorimaging.

Protein localisation in yeast and quantitative live cell imaging.
Fluorescent images were recorded with an applied precision PersonalDV microscope with a 60x oil immersion objective as stacks of 0.25 µm thick slices. Pictures from one figure panel are taken with the same imaging setup. Maximum intensity Z projections (Fig. 4a and S3a,b), sums of 5 slices (Fig. 4c) or single slices (Fig. 5a,c, S4a) of these stacks are shown. The contrast was linearly adjusted for each of the pictures. In Fig. 4a the GFP channel was deconvolved. Quantification was performed on original pictures prior to deconvolution using ImageJ software.

PAS intensity: average pixel brightness of the PAS; PAS area: average size of the PAS in pixel; cell intensity: average pixel brightness of the whole cell. Giant Ape1 assays were performed as previously described (Suzuki et al., 2013), using 3 hours of 250nM $CuSO_4$ induction and 1 hour of 300nM rapamycin treatment.

Peptide array analysis. The consensus phosphorylation sequence for native Atg1 complex was determined by screening a positional scanning peptide library as previously described (Mok et al., 2010). The peptide library consisted of 201 mixtures, each with the general sequence Y-A-X-X-X-X-X-S/T-X-X-X-X-A-G-K-K(biotin), where X is an equimolar mixture of the 17 amino acids excluding Cys, Ser and Thr, and S/T is an equimolar mixture of Ser and Thr. For each mixture, one of the X positions was fixed as either one of the 20 unmodified amino acids, phosphothreonine, or phosphotyrosine. Three additional peptides were included that substituted Ser, Thr or Tyr at the central phosphorylation site position to assess phosphoacceptor preference. Peptides (50 μM in 50 mM HEPES, pH 7.4, 1 mM EGTA, 15 mM $MgCl_2$, 0.1 mM Na_3VO_4, aprotinin, leupeptin, pepstatin, and 0.1% tween) were incubated with purified Atg1 complex and ATP (50 μM including 0.04 μCi/μl [γ-^{33}P]ATP) in a 1536 well plate (2 μl reaction volume per well) for 2 hr at 30 ºC. Following incubation, 200 nl aliquots of each reaction were transferred to a streptavidin membrane (SAM2 biotin capture membrane, Promega), which was washed as described, dried, and exposed to a phosphor screen. Spot intensities were quantified (Quantity One software, Bio-Rad), and data from two separate runs were normalized as described, averaged, and arranged into a position-specific scoring matrix (PSSM). Candidate Atg1 phosphorylation sites were identified by scanning the yeast proteome with the program Scansite (Obenauer et al., 2003) using the PSSM as an input. The heat map was generated with the program Maple Tree using the log-transformed PSSM.

SILAC labelling, HTBeaq tandem affinity purification and Mass spectrometric analysis. SILAC labeling, protein purification and mass spectrometric analysis is based on methods described elsewhere (Reiter et al., 2012). Regulated protein phosphorylation events were determined as described in (Reiter et al., 2012) (see supplemental information) with modifications: For phospho-mapping purified proteins were digested using different proteases (5% w/w of the estimated protein amount): Trypsin (recombinant, proteomics grade,

Roche) digest was performed overnight at 37°C, chymotrypsin digest (both sequencing grade, Roche) was performed at 25°C for 4 hours. MS analysis was performed using a reversed phase nano-HPLC (Ultimate 3000, Dionex, Sunnyvale CA, USA) directly coupled to an LTQ OrbitrapVelos mass spectrometer (Thermo Fisher Scientific) via a nanoelectrospray ion source (Proxeon).

A phosphosite probability (calculated using phosphoRS 2.0) of 75% or higher was considered as confidently localized. Results were divided in lists of highly confident localized and non-localized (< 75% probability) phosphorylation sites (Supplementary Table S1). Unphosphorylated peptides are also listed in Supplementary Table S1. Additionally, results were also filtered at the XCorr values to an FDR of 5% on the peptide level. These data were similarly processed as described above, except that all phosphopeptide assignments were also validated manually and SILAC quantification was performed by manual peak integration of extracted ion chromatograms (LTQ-FT data).

***In vitro* phosphorylation and mass spectrometric identification of synthetic peptides.** Peptide sequences to be synthesized were selected using *in silico* digestion of candidate substrate proteins with trypsin. Proteotypic tryptic peptides of ≤20 amino acids in length were selected and synthesized in their crude form at 60 nmoles/peptide (JPT Peptide Technologies). If there were sequence sections longer than 20 amino acids, then a lysine was added artificially to the end of sequences to allow for synthesis. To solubilize, the peptides were reconstituted in 20% acetonitrile: 80% deionized H_2O and sonicated by floating the 96-well plates in a sonicator water bath for 10 minutes. Peptides were pooled in no more than 96 peptides per pool and reconstituted such that the concentration of each peptide in the pool was 50 pmoles/µl which is 10x the final concentration for our *in vitro* phosphorylation reactions. For *in vitro* phosphorylation, purified soluble Atg1 kinase and soluble Atg1-kinase dead (Atg1-D211A) were added to a reaction tube with a volume of 65 µl and a final concentration of 50 mM Tris, 100 mM NaCl, 5 mM $MgCl_2$, 0.2 mM EDTA, and 5% glycerol (reagents from Sigma-Aldrich) at a pH of 7.5. Adenosine triphosphate (Roche Applied Sciences) was added to a final concentration of 250 µM and the final concentration of each peptide in solution was 5 µM. Reactions were performed at 30^0C with shaking at 1200 rpm in parallel where no more than 200 peptides were included in any reaction tube. Time points were taken from each reaction tube at 5 minutes, 30 minutes, and 60 minutes (20

µl for each time point). Phosphorylation was quenched by denaturation of the kinase with 5 M urea in 0.1 M ammonium bicarbonate directly on ice. Samples were subsequently reduced using 10 mM *tris*(2-carboxyethyl)phosphine at a final pH 7.5 for 30 min and cysteines were alkylated using freshly prepared 20 mM iodoacetamide at a final pH 7.5 for 30 minutes in the dark (reagents from Sigma-Aldrich). Samples were then enzymatically cleaved using 1:100 trypsin (Promega) at 35 ºC overnight. After cleavage, peptides were acidified using 50% formic acid to a final pH 2.5-2.7 and desalted using reversed-phase silica-based chromatography columns (Waters). The final elution buffer after the peptides were washed on the columns was 40% acetonitrile: 0.1% formic acid in HPLC grade water (binding buffer). PHOS-Select™ Iron Affinity Gel (Sigma-Aldrich) was used for immobilized metal affinity chromatograph (IMAC) to enrich for phosphorylated peptides at a ratio of 30 µl of beads per sample. Binding was performed for 1 hour at room temperature with slow end-over-end rotation. After phosphopeptide binding, beads were packed onto self-made StageTips as described (Villén and Gygi, 2008) and desalting and elution washing steps were performed as described. Finally, after elution and removal of the elution buffer by evaporation in a heated vacuum centrifuge (SpeedVac, Labconco Acid-Resistant CentriVap Concentrator), peptides were reconstituted in 40 µL deionized water with 0.1% formic acid. Each time point was injected into a Thermo Scientific nano HPLC in technical duplicates for mass spectrometric analysis at a volume of 5 µL that was coupled to an LTQ-Orbitrap XL (Thermo Scientific) equipped with a nanoelectrospray ion source (Thermo Scientific). Data-dependent acquisition was performed with an automatic switch between MS and MS/MS scans. High-resolution MS scans were acquired in the Orbitrap (60,000 FWHM, target value 106) to monitor peptide ions in the mass range of 350–1,650 m/z, followed by collision-induced dissociation MS/MS scans in the ion trap (minimum signal threshold 150, target value 104, isolation width 2 m/z) of the five most intense precursor ions. Dynamic exclusion was used with a 10 second interval applied after the fragmentation of intense precursors. Singly charged ions and ions with unassigned charge states were excluded from triggering MS2 events. The raw data files were converted to the mzXML format and peptides were searched using a Sorcerer server employing the SEQUEST algorithm against the Saccharomyces Genome Database (www.yeastgenome.org) containing both forward and reverse sequences used for false discovery rate determination. For *in silico* digestion, trypsin was used as the protease and was assumed to cleave after lysine (K) and

arginine (R) unless followed by proline (P). Two missed cleavage sites and one non-tryptic terminus were allowed per peptide. The precursor ion tolerance was set to 50 parts per million (ppm), and fragment ion tolerance was set to 0.5 dalton. The data were searched allowing phosphorylation (+79.9663 daltons) of serine, threonine, and tyrosine as a variable modification and carboxy-amidomethylation of cysteine (+57.0214 daltons) residues as a fixed modification. Additionally, the final search results were filtered above a 1% false discovery rate. Phosphorylation sites were scored using the PTMProphet algorithm available as part of the Trans-Proteomic Pipeline 4.6.1. and scores higher than 0.7 were considered correct phosphorylation site identifications. Results were quantified on a chromatographic level using the MS1-filtering routine available in the freely available Skyline version 1.4 (Schilling et al., 2012).

Supplemental References

Gruhler, A., Olsen, J.V., Mohammed, S., Mortensen, P., Faergeman, N.J., Mann, M., and Jensen, O.N. (2005). Quantitative phosphoproteomics applied to the yeast pheromone signaling pathway. Mol Cell Proteomics *4*, 310–327.

Kijanska, M., Dohnal, I., Reiter, W., Kaspar, S., Stoffel, I., Ammerer, G., Kraft, C., and Peter, M. (2010). Activation of Atg1 kinase in autophagy by regulated phosphorylation. Autophagy *6*, 1168–1178.

Klionsky, D.J. (2007). Monitoring autophagy in yeast: the Pho8Delta60 assay. Methods Mol Biol *390*, 363–371.

Kraft, C., Kijanska, M., Kalie, E., Siergiejuk, E., Lee, S.S., Semplicio, G., Stoffel, I., Brezovich, A., Verma, M., Hansmann, I., et al. (2012). Binding of the Atg1/ULK1 kinase to the ubiquitin-like protein Atg8 regulates autophagy. Embo J.

Mok, J., Kim, P.M., Lam, H.Y.K., Piccirillo, S., Zhou, X., Jeschke, G.R., Sheridan, D.L., Parker, S.A., Desai, V., Jwa, M., et al. (2010). Deciphering protein kinase specificity through large-scale analysis of yeast phosphorylation site motifs. Sci Signal *3*, ra12.

Obenauer, J.C., Cantley, L.C., and Yaffe, M.B. (2003). Scansite 2.0: Proteome-wide prediction of cell signaling interactions using short sequence motifs. Nucleic Acids Res. *31*, 3635–3641.

Reiter, W., Anrather, D., Dohnal, I., Pichler, P., Veis, J., Grøtli, M., Posas, F., and Ammerer, G. (2012). Validation of regulated protein phosphorylation events in yeast by quantitative mass spectrometry analysis of purified proteins. Proteomics *12*, 3030–3043.

Romanov, J., Walczak, M., Ibiricu, I., Schüchner, S., Ogris, E., Kraft, C., and Martens, S. (2012). Mechanism and functions of membrane binding by the Atg5-Atg12/Atg16 complex during autophagosome formation. Embo J.

Schilling, B., Rardin, M.J., MacLean, B.X., Zawadzka, A.M., Frewen, B.E., Cusack, M.P., Sorensen, D.J., Bereman, M.S., Jing, E., Wu, C.C., et al. (2012). Platform-independent and Label-free Quantitation of Proteomic Data Using MS1 Extracted Ion Chromatograms in Skyline: APPLICATION TO PROTEIN ACETYLATION AND PHOSPHORYLATION. Molecular & Cellular Proteomics *11*, 202–214.

Sikorski, R.S., and Hieter, P. (1989). A system of shuttle vectors and yeast host strains designed for efficient manipulation of DNA in Saccharomyces cerevisiae. Genetics *122*, 19–27.

Suzuki, K., Akioka, M., Kondo-Kakuta, C., Yamamoto, H., and Ohsumi, Y. (2013). Fine mapping of autophagy-related proteins during autophagosome formation in Saccharomyces cerevisiae. J Cell Sci.

Villén, J., and Gygi, S.P. (2008). The SCX/IMAC enrichment approach for global phosphorylation analysis by mass spectrometry. Nat Protoc *3*, 1630–1638.

Zuzuarregui, A., Kupka, T., Bhatt, B., Dohnal, I., Mudrak, I., Friedmann, C., Schüchner, S., Frohner, I.E., Ammerer, G., and Ogris, E. (2012). M-Track: detecting short-lived protein-protein interactions in vivo. Nat Meth *9*, 594–596.
